# Regulation of human cortical interneuron development by the chromatin remodeling protein CHD2

**DOI:** 10.1038/s41598-022-19654-y

**Published:** 2022-09-17

**Authors:** E. M. A. Lewis, G. Chapman, K. Kaushik, J. Determan, I. Antony, K. Meganathan, M. Narasimhan, P. Gontarz, B. Zhang, K. L. Kroll

**Affiliations:** Department of Developmental Biology, Washington University School of Medicine, Saint Louis, MO 63110 USA

**Keywords:** Neurogenesis, Developmental neurogenesis, Embryonic stem cells, Neural stem cells, Epigenetic memory, Development of the nervous system, Epigenetics in the nervous system, Neurogenesis, Embryonic stem cells, Epigenetic memory, Neural stem cells

## Abstract

Mutations in the chromodomain helicase DNA binding protein 2 (*CHD2*) gene are associated with neurodevelopmental disorders. However, mechanisms by which CHD2 regulates human brain development remain largely uncharacterized. Here, we used a human embryonic stem cell model of cortical interneuron (hcIN) development to elucidate its roles in this process. We identified genome-wide CHD2 binding profiles during hcIN differentiation, defining direct CHD2 targets related to neurogenesis in hcIN progenitors and to neuronal function in hcINs. CHD2 bound sites were frequently coenriched with histone H3 lysine 27 acetylation (H3K27ac) and associated with high gene expression, indicating roles for CHD2 in promoting gene expression during hcIN development. Binding sites for different classes of transcription factors were enriched at CHD2 bound regions during differentiation, suggesting transcription factors that may cooperatively regulate stage-specific gene expression with CHD2. We also demonstrated that CHD2 haploinsufficiency altered CHD2 and H3K27ac coenrichment on chromatin and expression of associated genes, decreasing acetylation and expression of cell cycle genes while increasing acetylation and expression of neuronal genes, to cause precocious differentiation. Together, these data describe CHD2 direct targets and mechanisms by which CHD2 prevents precocious hcIN differentiation, which are likely to be disrupted by pathogenic *CHD2* mutation to cause neurodevelopmental disorders.

## Introduction

Disruption of the processes that regulate fetal brain development can contribute to the etiology of neurodevelopmental disorders (NDDs), including autism spectrum disorder (ASD) and epileptic encephalopathies, the latter of which are a group of pediatric onset epilepsies with poor prognoses^1–3^. Many of these disorders arise, at least in part, from altered development of excitatory and inhibitory neurons in the cerebral cortex, where GABAergic cortical interneurons (hcINs) and glutamatergic excitatory projection neurons normally interact to modulate the balance of excitatory and inhibitory neuronal inputs to enable proper cortical function^4–6^. Transcription factors (TFs) and chromatin modifying proteins act together to control gene regulatory networks that underlie the specification and differentiation of both hcINs and cortical excitatory neurons. Mutations in genes encoding chromatin modifying proteins are over-represented among pathogenic contributors to NDDs, further demonstrating the critical nature of these proteins during neurodevelopment^7–9^. However, there is little known about the role of many of these proteins in and how their mutation alters neurodevelopment.

CHD2 is part of the nine-member chromodomain helicase DNA-binding (CHD) family of chromatin remodeling proteins. CHD family proteins are involved in a range of developmental processes, while mutations in several *CHD* family members cause human diseases including NDDs^10,11^. These proteins all contain chromodomains, which mediate their association with chromatin, and ATP-dependent helicase domains, which can remodel nucleosomes, while CHD1 and CHD2 also contain domains for direct binding to DNA^11,12^.

While the direct targets of CHD2 and mechanisms of action during brain development remain unknown, CHD2 has been shown to co-bind the genome together with cell type-specific transcription factors, including MyoD during myocyte differentiation^13^ and OCT3/4 in mouse embryonic stem cells^14^. Likewise, in our prior work we showed that CHD2 could co-bind the genome with the homeodomain transcription factor NKX2.1 at several target genes during specification of human ESCs (hESCs) to medial ganglionic eminence-like progenitors (hMGEs) and during their differentiation into hcINs^15^. CHD2 has also been shown to enhance deposition of the variant histone H3.3; which is associated with hyperdynamic chromatin enrichment at the promoter of genes that regulate cell-type specific developmental programs^13,14,16,17^, and is generally associated with poised or active histone modifications^14,17^. Finally, in addition to roles in regulating chromatin state to control gene expression, CHD2 has also been shown to modulate DNA damage repair by increasing nucleosome spacing and interaction with PARP1, potentially via promoting H3.3 incorporation into nucleosomes^18^.

Requirements for CHD2 activity during brain development have been studied in other developmental contexts, primarily in mouse models. Two CHD2 deficient mouse models displayed evidence of abnormal neural development although, similar to prior work in human cellular models, neither recapitulated a human epilepsy phenotype^16,19^. Other studies characterizing mouse models of CHD2 deficiency did not report neurological abnormalities and instead identified spinal, blood, and renal abnormalities^2,20–22^. We also previously demonstrated delayed maturation of hESC-derived human cortical interneurons following either shRNA-based knockdown of *CHD2* or CRISPR-based biallelic *CHD2* knockout, with these hcINs exhibiting subsequent electrophysiological abnormalities, while human cortical excitatory neurons displayed no electrophysiological abnormalities^15^.

Building upon these studies, we sought to elucidate the role of and mechanisms by which CHD2 regulates hcIN development. To do this, we first defined genomic locations bound by CHD2 during hESC specification into hMGE progenitors and their subsequent differentiation into hcINs, as well as examining corresponding changes in chromatin state at these regions during specification and differentiation. To understand requirements for CHD2 action we also manipulated CHD2 expression, generating an hESC line with heterozygous *CHD2* mutation, which mimics the haploinsufficiency seen in many patients heterozygous for pathogenic *CHD2* mutation or deletion. This work revealed cis-sequence features underlying CHD2 binding, chromatin state changes that occurred at these locations during differentiation, and key aspects of these developmental programs that were disrupted by *CHD2* haploinsufficiency. Together, our work here has defined direct targets and mechanisms of action by which CHD2 controls hcIN development and has determined how these are disrupted under conditions of *CHD2* haploinsufficiency, providing a framework for understanding why the *CHD2* gene is among those least tolerant to sequence variation in the human genome^3^.

## Results

### Expression of genes associated with CHD2 binding during hcIN differentiation

**Figure 1:**
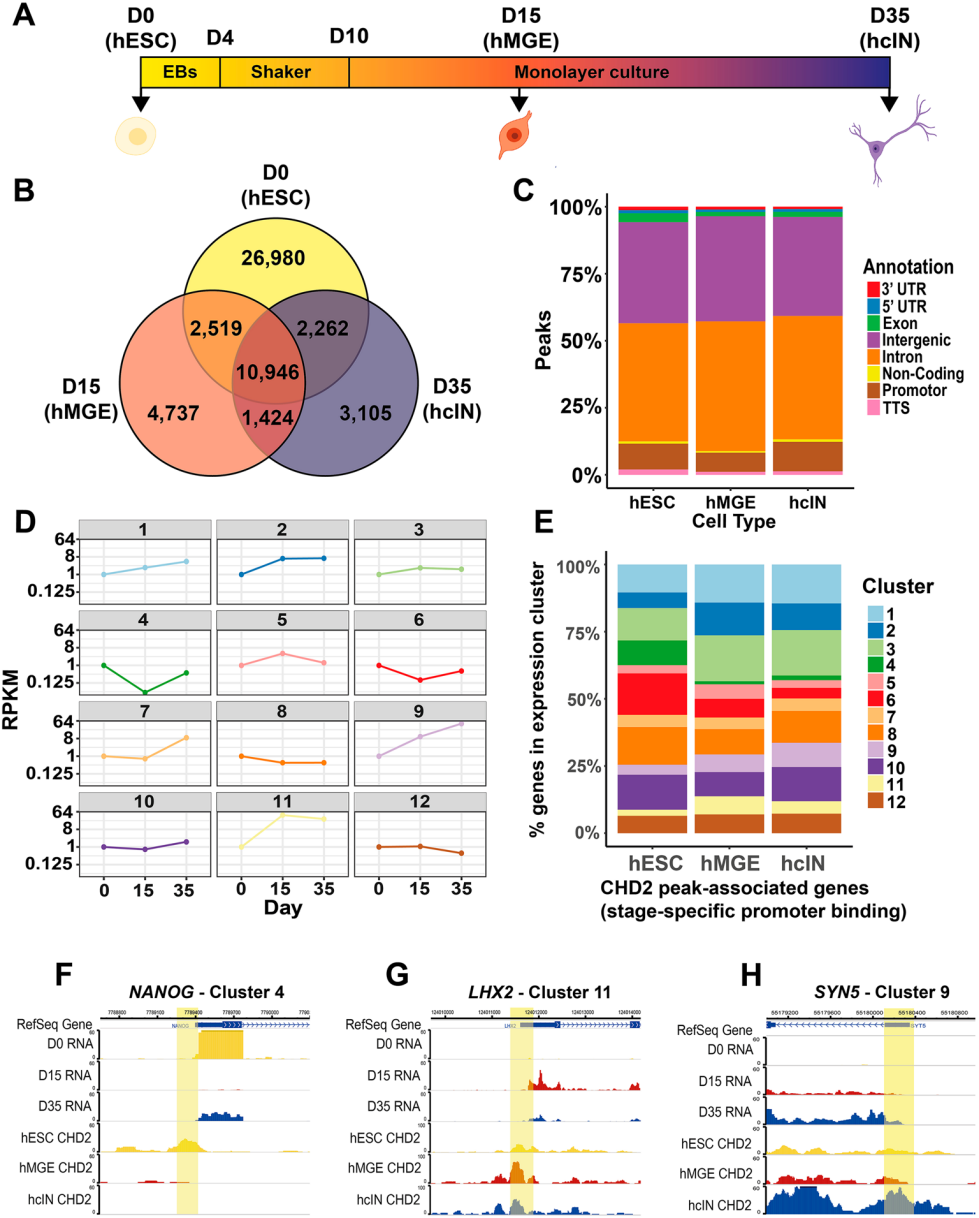
Genome-wide interrogation of CHD2 binding during hcIN differentiation from hESCs. (**A**) Differentiation scheme used to produce human cortical interneurons (hcINs) from human embryonic stem cells (hESCs) in vitro. Samples were collected and analyzed at day 0 (D0, hESCs), day 15 (D15; medial ganglionic eminence-like progenitors, hMGEs), and day 35 (D35, hcINs). (**B**) Number of peaks bound by CHD2 at each time point assayed, including stage-specific 'unique' peaks (D0, D15, and D35) and peaks present at multiple time points. (**C**) Genomic annotation of CHD2 bound peaks identified in hESCs, hMGEs, and hcINs, for all peaks present at each cell stage. (**D**) Examples of gene expression during differentiation of wild type (WT) hESCs into hcINs. 12 gene expression profile clusters were generated, with all genes in each cluster having similar expression trends; each example expression profile represented here corresponds to the cluster centroid values, assuming a D0 RPKM value of 1. (**E**) Distribution of genes with stage-specific, ‘unique’ CHD2 binding at the promotor region across the 12 WT gene expression clusters. (**F**–**H**) Epigenome Browser views for example genes in cluster 4 (**F**, *NANOG*), 11 (**G**, *LHX2*) and 9 (**H**, *SYN5*), showing RNA expression and CHD2 binding from hESC (D0) to hcIN (D35). Promoter proximal CHD2 peaks are indicated as yellow highlights.

To understand how CHD2 might regulate hcIN differentiation, we assessed its genome-wide binding profile at day 0 (in hESCs), day 15 (in hMGEs), and day 35 (in hcINs) (Fig. 1A; Table S1), observing more peaks in hESCs (42,707), by comparison with hMGEs (19,626), or hcINs (17,737) (Fig. 1B). To assess how CHD2 peak locations changed during hcIN specification and differentiation, peaks were divided into those unique to one time point or present (shared) at two or more time points. Almost half of all hESC peaks (26,980) were unique to this time point. A similarly large number of peaks (10,946) were shared between all three cell states profiled, while smaller numbers of peaks were unique to hMGEs (4737) or hcINs (3105) (Fig. 1B). These peaks may be associated with roles that CHD2 performs specifically during hMGE specification or hcIN differentiation.

Since regulatory proteins often bind both promoter-proximal and distal regulatory elements, we next investigated genomic annotations of CHD2-bound regions. During specification and differentiation, we found no major differences between annotations of CHD2 bound peaks, with a large number being either in introns or intergenic space, some of which may overlap cis-regulatory elements, while a smaller fraction were at gene promoters (Fig. 1C).

We hypothesized that stage specific CHD2 binding could be associated with correspondingly increased expression of the nearest associated gene. To investigate this, we assessed how expression of CHD2-associated genes changed during differentiation. Using our previously derived RNA-sequencing data for hESCs (day (D) 0), hMGEs (D15), and hcINs (D35) (Table S2), we clustered all genes with a greater than 1.5-fold change in gene expression either between D0-D15 or between D15–D35, to define 12 clusters of genes with similar expression profiles (Fig. 1D, Sup. Fig. S1). To validate the use of this clustering approach we examined 3 key markers of hMGE progenitors (*ASCL1, DLX2* and *NKX2.1*, Sup. Fig. S1), finding that they belonged to cluster 11, which contains genes that peak at day 15 as was expected for these hMGE markers. We also examined 3 key markers of hcINs (*SST*, *GAD1* and *GAD2*, Sup. Fig. S1) and found that they were assigned to cluster 9, which showed increased expression across differentiation as was expected for hcIN markers.

To focus on the highest-confidence regulatory element-to-gene associations, we focused on CHD2 peaks within 2 kb up- or downstream of the transcription start site (TSS; Table S1) and those peaks bound by CHD2 only at one stage (e.g. CHD2 bound uniquely in hESCs, but not in hMGEs or hcINs) and identified the expression cluster corresponding to each CHD2-associated gene. The enriched expression clusters differed, depending upon the stage of CHD2 binding examined. For example, CHD2 bound genes in hESCs were enriched for clusters 4 (dark green), 6 (red), and 8 (orange), each of which show decreased expression from D0-D15, during hESC pluripotency exit and hMGE specification (Fig. 1D,E). By contrast, CHD2 bound genes in hMGEs and hcINs were enriched for expression clusters 1 (light blue), 2 (dark blue), 3 (light green), 9 (light purple) and 11 (yellow), all of which increase in expression during hMGE specification and hcIN differentiation (Fig. 1D,E). Examples of the CHD2 binding and expression profiles for genes in some of these clusters are shown (Fig. 1F–H). These findings support our hypothesis that temporally restricted CHD2 binding during hcIN differentiation is associated with increased gene expression.

### CHD2 associates with suites of genes involved in stage-related developmental processes

**Figure 2:**
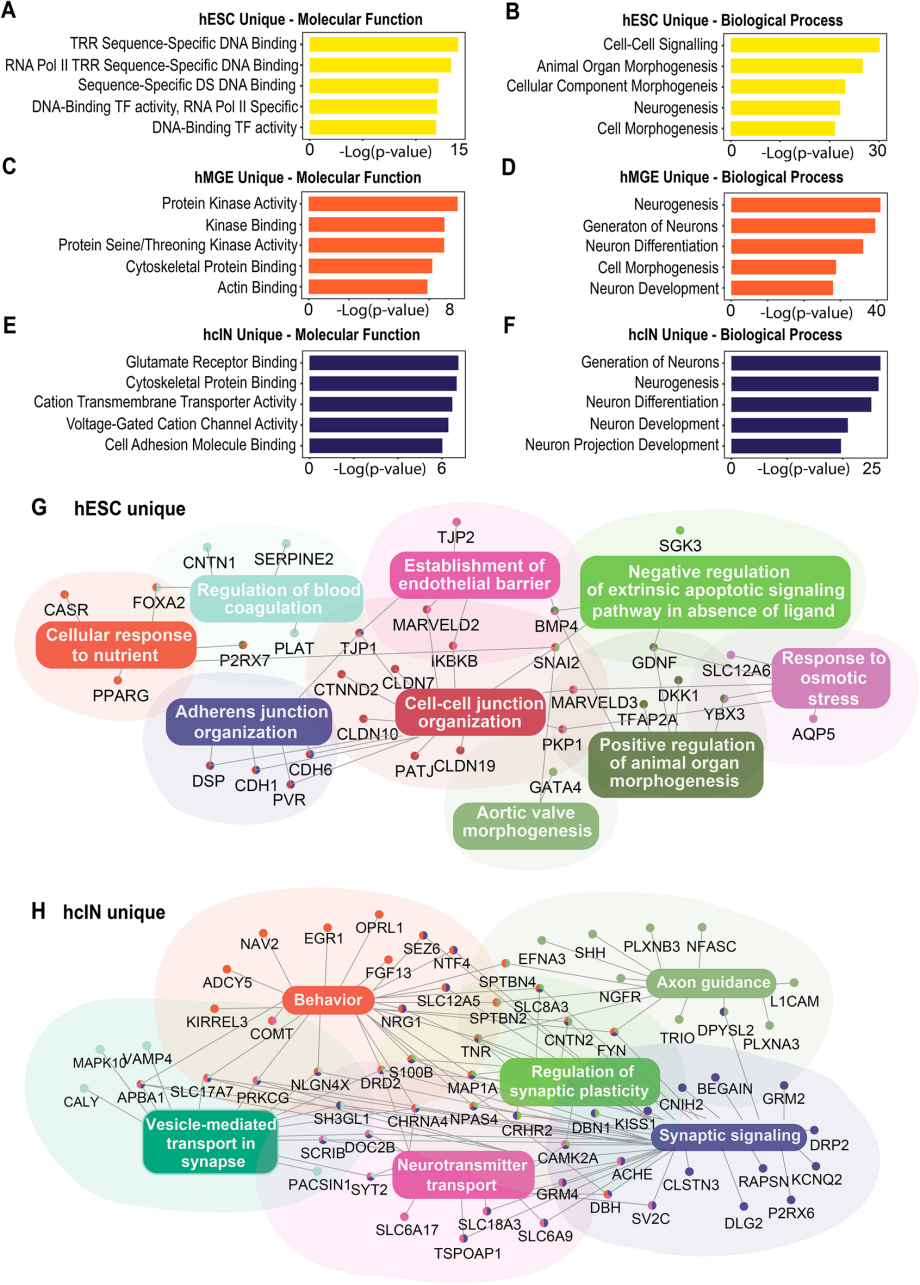
Analysis of genes associated with CHD2 binding during hcIN differentiation. (**A**–**F**) Gene ontology (GO) enrichment analysis of genes with a promoter proximal CHD2 peak uniquely in (**A**, **B**) hESCs, (**C**, **D**) hMGEs, or (**E**, **F**) hcINs. − Log10 *p* values for top enriched terms are shown. (**G**, **H**) Gene networks generated from a single representative GO term. In (**G**), the hESC ‘unique’ CHD2-bound gene network was generated by focusing on intracellular junction-related genes. In (**H**), the hcIN ‘unique’ CHD2-bound gene network was generated by focusing on synapse-related genes.

To explore the identity of these CHD2-associated genes, we next performed gene enrichment analysis. Specifically, genes associated with at least one CHD2 bound peak that was proximal to the promoter and only bound at one stage (i.e. hESC, hMGE, or hcIN 'unique' peaks) were used for analysis. Terms associated with genes with hESC-unique CHD2 bound peaks included ‘cell–cell signaling’ and ‘morphogenesis’ (Fig. 2A,B, Table S3). Genes with at least one CHD2-associated peak unique to hMGEs or hcINs were instead predominantly associated with terms related to neurodevelopment (Fig. 2C–F, Table S3). For example, terms associated with hMGE-unique CHD2 peaks included ‘neurogenesis’ (Fig. 2C,D, Table S3), while terms associated with hcIN-unique CHD2 peaks were associated with more mature neurons, including ‘voltage-gated cation channel activity’ and ‘neuron projection development’ (Fig. 2E,F; Table S3). By comparison with these stage-specific binding events, terms associated with ‘all’ peaks (shared or unique) bound by CHD2 in hMGEs or hcINs were associated with similar processes, but the associations were less pronounced (Table S3).

Pathogenic mutation of CHD2 is known to contribute to NDDs, including ASD and epilepsy, while many ASD- and epilepsy-associated genes are likewise important for neural development and neuronal function. We therefore compared genes that were bound by CHD2 at each developmental time point in our experimental scheme with known ASD and epilepsy genes. From a list of 102 ASD risk-associated genes^23^, 53 (52%) were bound by CHD2 at some point during hcIN differentiation, while many (34) of these genes were bound by CHD2 in hESCs, hMGEs, and hcINs (Supplemental Fig. S2; ASD). Most of these genes had low expression in hESCs and were highly expressed in hcINs, congruent with the involvement of many ASD-associated genes in neuronal differentiation or maturation (Supplemental Fig. S2). In comparisons with 536 epilepsy-related genes^24^, 57 (11%) were also bound by CHD2 at some point during hcIN differentiation, and many (32) of these were likewise CHD2 bound in hESCs, hMGEs, and hcINs (Supplemental Fig. S2; Epilepsy).

We next performed network analysis of the genes associated with CHD2 peaks unique to hESCs, hMGEs, or hcINs. Themes output from this analysis were congruent with the previously identified GO terms (Table S3), and one representative high-scoring theme for each stage of differentiation was selected for further analysis. For CHD2 bound genes in hESCs, a major theme was related to intracellular junctions and epithelial identity, and included genes such as *MARVELD2/3*, *CLDN7/10/19*, and *CDH1/6* (Fig. 2G). For CHD2 bound genes in hMGEs, this theme was related to neuroectoderm development and included genes such as *GBX2*, *SMAD2/4*, *ZIC1/3*, and *BMP7* (Supplemental Fig. S3). For CHD2 bound genes in hcINs, this theme was related to synapses, and included genes such as *CHRNA4*, *SLC17A7*, *NPAS4*, and *CNTN2* (Fig. 2H). Together, this indicates that CHD2 may be involved with maintaining proper expression of genes required for cell adhesion and cell cycle progression during the transition from pluripotency through early neural fate acquisition, as well as those required for later neuronal specification, differentiation, and maturation, further confirming a relationship between the stage of CHD2 binding and expression and activity of the associated genes.

### CHD2 peaks in hMGEs and hcINs contain binding motifs for transcription factors associated with hcIN development

**Figure 3:**
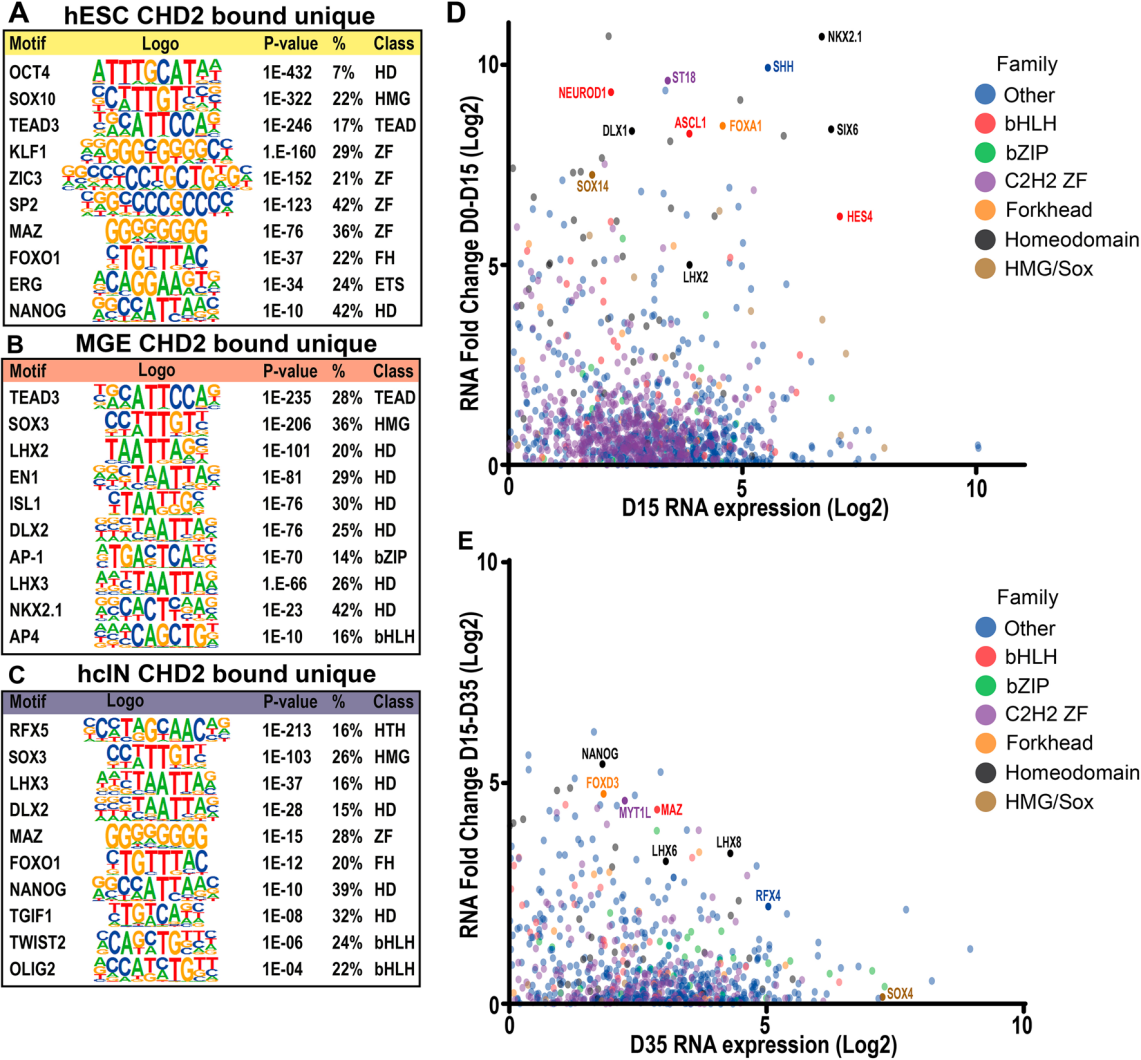
Analysis of candidate TFs that may co-bind the genome with CHD2 during hcIN differentiation. (**A**–**C**) Predicted transcription factor (TF) binding motifs under CHD2 peaks unique to (**A**) hESCs, (**B**) hMGEs and (**C**) hcINs. Adjusted p-values, the percentage of peaks with each TFBS, and the TF class are shown (HD = homeodomain, ZF = Zinc finger, FH = forkhead). (**D**) Expression changes of up-regulated TFs from hESC (D0) to hMGE (D15). Only TFs with a RPKM value greater than 1 (i.e. log2 RPKM > 0) and a positive fold change from D0 to D15 (i.e. log2 FC > 0) are shown. (**E**) Expression changes of up-regulated TFs from hMGE (D15) to hcIN (D35). Only TFs with a RPKM value greater than 1 (i.e. log2 RPKM > 0) and a positive fold change from D15 to D35 (i.e. log2 FC > 0) are shown.

Since CHD2 can co-regulate gene expression with cell type-specific transcription factors (TFs)^13–15^, we next explored the sequence enrichment for TF binding sites (TFBSs) under CHD2 peaks. As in prior analyses, we focused on CHD2-bound peaks that were bound in a stage-specific manner (“unique” peaks, including all peaks regardless of the distance to the nearest TSS), as these may represent regulatory elements specific to aspects of hcIN specification or differentiation. At the hESC stage, a range of TFBS were present at unique CHD2 binding sites (Fig. 3A, Table S4). Many of these transcription factors (e.g. OCT4, NANOG, and ZIC3) play roles in pluripotency and are highly expressed in hESCs.

At the hMGE stage, CHD2 bound sites continued to be enriched for TEAD binding motifs (Fig. 3B, Table S4); correspondingly, TEAD factors were highly expressed at both the hESC and hMGE stages (Supplemental Fig. S4; TEAD). CHD2 peaks specific to hMGE progenitors were also enriched for a number of homeodomain TFBS including those recognized by LHX2, DLX2, and NKX2.1, all of which play roles in hcIN development (Fig. 3B, Table S4). To further examine the potential relevance of these TFBS, we assessed the relative expression of all TFs from D0 to D15 of differentiation and annotated a subset of TFs that exhibited increased expression at D15 relative to D0 (Fig. 3D, Table S5). This analysis further highlighted multiple homeodomain TFs identified by the TFBS analysis, including NKX2.1, DLX1, SIX6 and LHX2, while further examination demonstrated that expression of many of these TFs is highest in hMGEs, relative to hESCs and hcINs (Supplemental Fig. S4; Homeodomain). These results suggest that CHD2 binding is associated with sites for binding by TFs that are important to specify hMGE progenitors.

Performing a similar TFBS analysis at the hcIN stage demonstrated enrichment for homeodomain, basic helix loop helix (bHLH), and Regulatory Factor binding to the X-box (RFX) TF binding motifs (Fig. 3C, Table S4). We found that, while many of these TFs were highly expressed at D35 (Fig. 3E, Table S5), their expression increased the most strongly from D0-D15 and then continued to increase D15–D35, suggesting roles in both specification and differentiation of hcINs (Fig. 3D,E, Supplemental Fig. S4, Table S5). Together, these data suggest that CHD2 may work with a range of TFs known to regulate the development of neurons or specifically cortical interneurons.

### CHD2 binding coenriches with H3K27ac in differentiating hcINs

After determining that CHD2 is frequently associated with genes with increasing expression during hcIN differentiation and is likely to co-localize at these sites with TFs that play important roles in this process, we next assessed the chromatin state of these putative regulatory elements. Since CHD2 has been shown to associate with poised or active chromatin^14,17^, we investigated three histone modifications associated with establishing these chromatin states: H3K4me3, which is enriched at active or bivalent promoters, H3K27me3, which enriches at repressive chromatin and bivalent promoters, and H3K27ac, which enriches at active enhancers.

**Figure 4:**
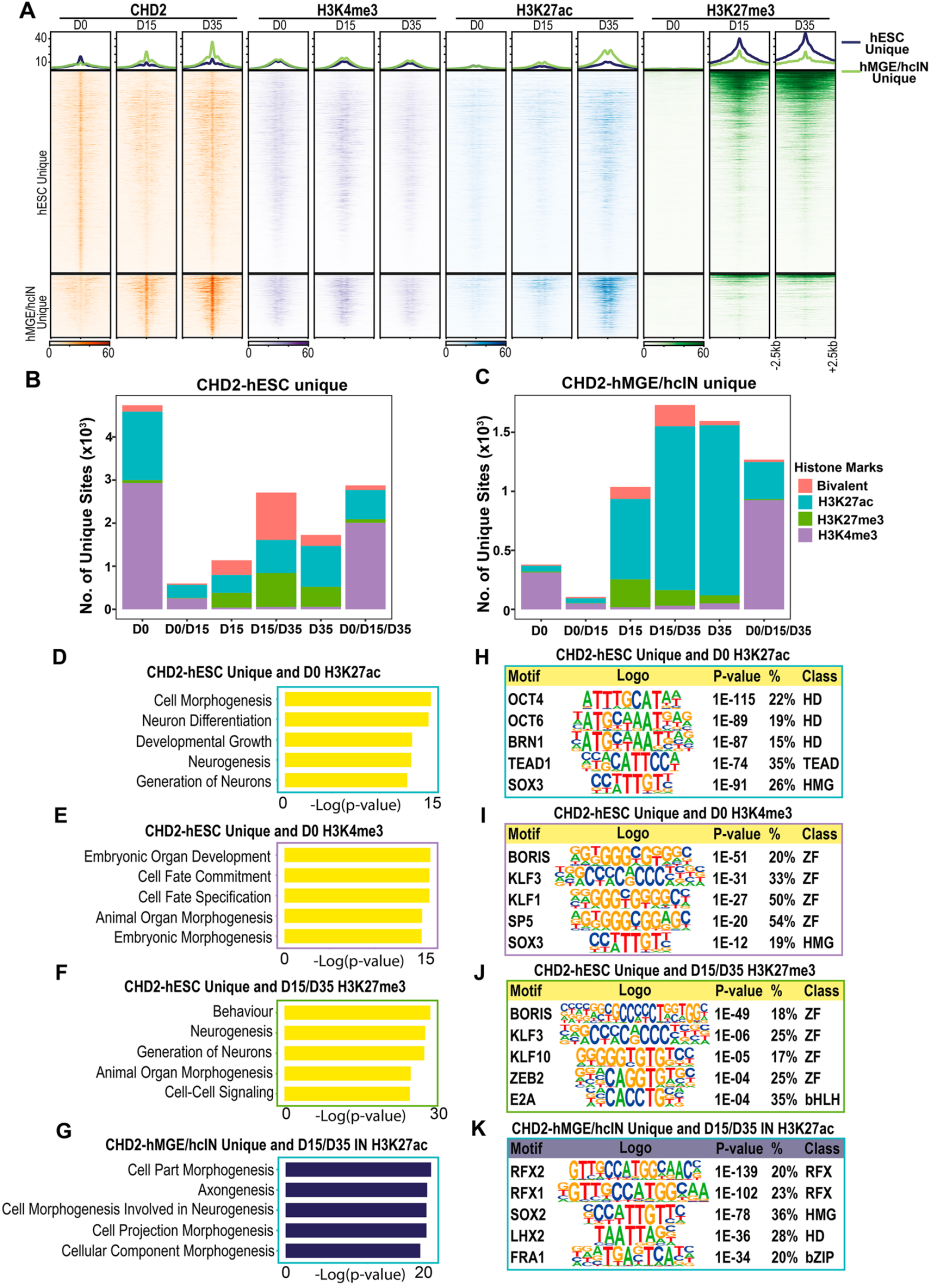
Histone modification states associated with CHD2 binding during hcIN differentiation. (**A**) Signal enrichment of H3K4me3, H3K27ac, and H3K27me3 modification around CHD2 bound peaks in hESCs, hMGEs, and hcINs. Line plots at top: averaged signal for CHD2 binding and histone modification in the hESC unique CHD2 peak cluster (upper panels) and the hMGE/hcIN unique peak cluster (lower panels), respectively. (**B**, **C**) Coenrichment of histone modifications with CHD2 bound peaks. For CHD2-bound peaks that were (**B**) unique to hESCs or (**C**) unique to hMGEs and/or hcINs but not hESCs, the number of unique overlapping peaks for each histone modification at the time indicated in days (**D**) is shown. Bivalent status was defined by the intersection between H3K4me3 and H3K27me3 peaks. (**D**–**F**) Gene ontology (GO) enrichment analysis of genes associated with CHD2 peaks unique to hESCs and enriched for (**D**) H3K27ac at D0, (**E**) H3K4me3 at D0, or (**F**) H3K27me3 at either/or D15 or D35. (**G**) Gene ontology (GO) enrichment analysis of genes with a CHD2 peak in hMGEs and/or hcINs and associated with H3K27ac at either/or D15 or D35, with p-values for top terms (expressed as -log10) shown. (**H**–**J**) Transcription factor binding site enrichment under CHD2 peaks unique to hESCs and enriched for (**H**) H3K27ac at D0, (**I**) H3K4me3 at D0, or (**J**) H3K27me3 at either/or D15 or D35. (**K**) Transcription factor binding site enrichment under CHD2 peaks in hMGEs and/or hcINs that were coenriched for H3K27ac at either/or D15 or D35.

By comparison with their chromatin state in hESCs, CHD2-bound, promoter-proximal (+/− 2 kb from the TSS) genomic regions exhibited increased H3K27ac coenrichment during hMGE specification and hcIN differentiation (Fig. 4A, Table S1). The peak subset that bound CHD2 in hMGEs and/or hcINs, but not in hESCs, showed the most pronounced CHD2 and H3K27ac coenrichment (Fig. 4A, Table S1). In examining all promoter-proximal genomic regions to which CHD2 binds in a non-temporally specific manner we also observed coenrichment with H3K4me3 at the hMGE stage and with H3K27ac at the hcIN stage, further suggesting an association between CHD2 binding and histone modifications associated with gene expression (Supplemental Fig. S5A).

To further examine the role of CHD2 binding in hcIN development we analyzed the chromatin state of all peaks (not just promoter-proximal) that were bound by CHD2 either uniquely in hESCs (Fig. 4B, Table S1) or in hMGEs and/or hcINs, but not hESCs (Fig. 4C, Table S1). Peaks uniquely bound by CHD2 in hESCs were most highly enriched for active histone modifications (H3K27ac or H3K4me3) in hESCs (Fig. 4B, D0), with a subset of these sites losing these active histone marks and/or gaining H3K27me3 during later stages of development. Peaks bound uniquely by CHD2 in hMGEs/hcINs instead coenriched predominantly for H3K27ac during differentiation (Fig. 4C; D15 and/or D35), having substantially less coenrichment with any histone marks at the hESC stage. However, it is important to note that, while both hESC-unique and hMGE/hcIN unique CHD2 peaks are generally enriched for H3K4me3 at the time of CHD2 binding, many of these peaks are coenriched for H3K4me3 during all stages of development (Fig. 4B,C D0/D15/D35). This trend is even more pronounced when examining sites where CHD2 is bound throughout development (Supplemental Fig. S5B, Table S1), suggesting that H3K4me3 coenrichment does not temporally relate to CHD2 binding or activity.

We also observed an increase in coenrichment of CHD2 with bivalent chromatin (carrying co-localized H3K27me3 and H3K4me3) at later stages of development, both at sites that are bound uniquely by CHD2 in hESCs and in hMGE/hcINs (Fig. 4B,C; D15 and/or D35). Examining all the sites of CHD2 peak coenrichment with bivalent chromatin, most of these sites were enriched for H3K4me3 at D0 and then gained the repressive H3K27me3 mark to become bivalent during differentiation (Fig. 4A, Supplemental Fig. S5C). This transition was more prevalent among the hESC-unique CHD2 peaks, suggesting that the gain of bivalency during differentiation involves a loss of CHD2 binding and a corresponding gain of the H3K27me3 mark (Fig. 4B,C; D15 and/or D35). Together, these findings support a model where CHD2 binding is associated with active regulatory elements and could contribute to maintaining an open/active chromatin state at these sites.

**Figure 5:**
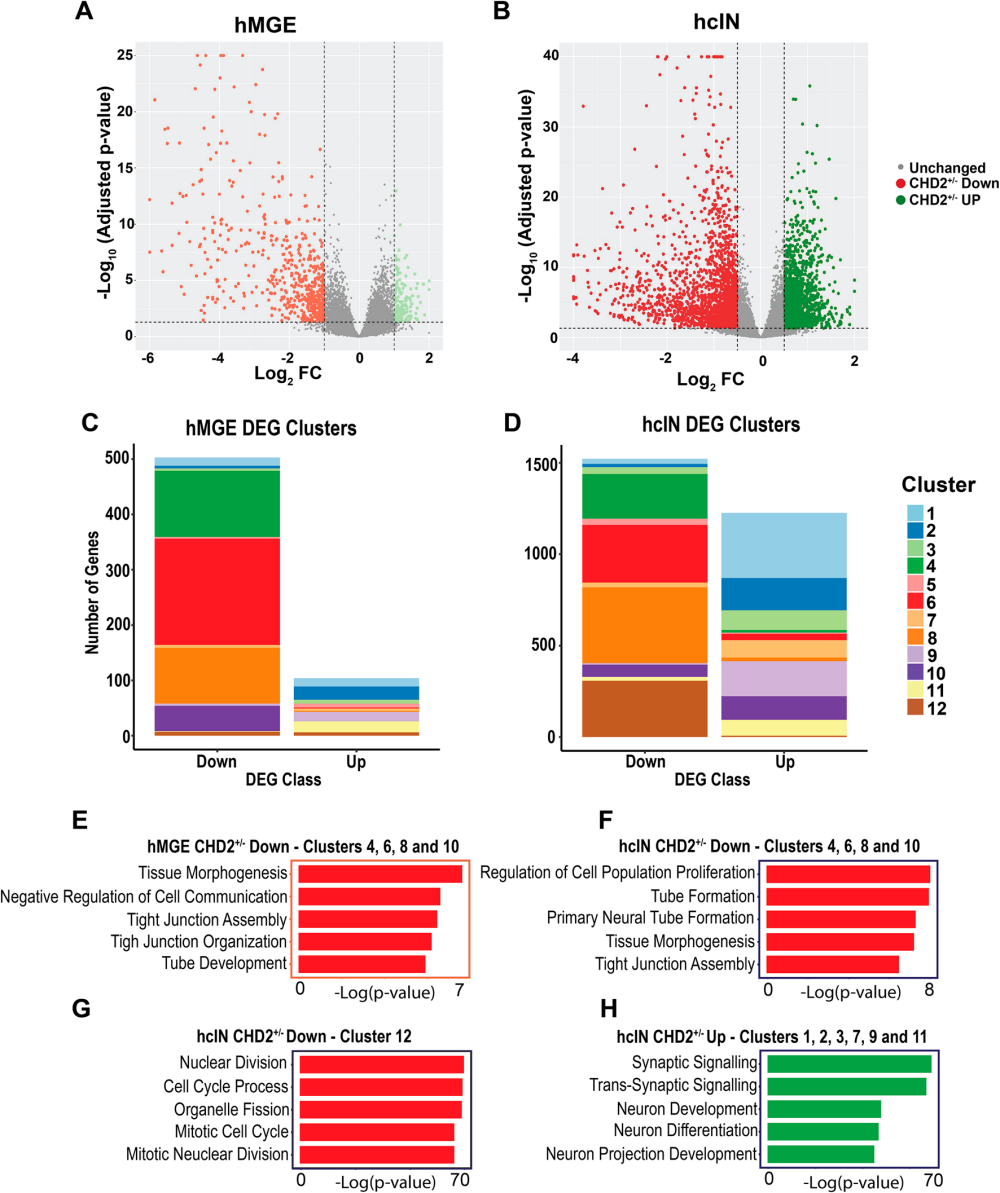
Gene expression changes in hMGEs and hcINs with CHD2 haploinsufficiency. (**A**, **B**) Gene expression differences (log2 FC) between wild type (WT) and mutant (CHD2^+/−^) in (**A**) hMGEs and (**B**) hcINs. Red: down-regulated genes in the mutant (CHD2^+/−^); green: up-regulated genes in the mutant (CHD2^+/−^). (**C**, **D**) Distribution across the 12 previously identified WT gene expression clusters for differentially expressed genes (DEGs) obtained in WT versus CHD2^+/−^ comparisons at the (**C**) hMGE and (**D**) hcIN stage. (**E**–**G**) Gene ontology (GO) enrichment analysis of DEGs down regulated in CHD2^+/−^ compared to WT models at the (**E**) hMGE or (**F**, **G**) hcIN stage. For hcINs, DEGs that mapped to WT expression (**F**) clusters 4, 6, 8 and 10, versus (**G**) cluster 12 were analyzed separately. (**H**) Gene ontology (GO) term analysis of DEGs that were upregulated in the CHD2^+/−^ compared to the WT model at the hcIN stage.

To gain a better understanding of which genes and pathways were associated with the changes in epigenetic state associated with CHD2 binding, we analyzed the top gene ontology (GO) terms for genes associated with subsets of CHD2 bound peaks with different histone modification states. For example, hESC-unique CHD2 bound peaks that coenriched with H3K27ac in hESCs were associated with genes related to neurodevelopment, including ‘Neuron Differentiation’ and ‘Neurogenesis’ (Fig. 4D, Table S6). By comparison, hESC-unique CHD2 peaks that co-localized with H3K4me3 were associated with general developmental genes, with top terms including ‘Embryonic Organ Development’ and ‘Cell Fate Commitment’ (Fig. 4E, Table S6). We also analyzed genes that were bound by CHD2 uniquely in hESCs but that gained the repressive H3K27me3 mark during neuronal differentiation (Fig. 4F, Table S7). The terms associated with these genes were similar to those obtained for the hESC-unique CHD2-bound peaks that co-localized with H3K4me3 and H3K27ac in hESCs, further suggesting that loss of CHD2 binding is associated with a transition from an active to a more repressive chromatin state at suites of developmental genes. CHD2-bound peaks in hMGEs/hcINs that coenriched with K3K27ac were also associated with genes related to neuronal differentiation; however, these terms were related to later neurodevelopmental processes, such as ‘Axongenesis’ (Fig. 4G, Table S6). Taken together, these results suggest that CHD2 binding is predominantly associated with histone marks denoting open and active chromatin and corresponding to genes involved in key developmental processes.

Since CHD2-bound peak subsets with different histone modification states were associated with developmentally distinct suites of genes, we also assessed the TFBS enrichment underneath these CHD2 peak subsets. This revealed distinct classes of TFBS enriched at each CHD2-bound peak subset. hESC-unique CHD2 peaks which co-localized with H3K27ac in hESCs were enriched for homeodomain and TEAD TFBS, including sites for OCT4 and TEAD1 (Fig. 4H, Table S7). hESC-unique CHD2 binding which co-localized with H3K4me3 in hESCs was instead associated with TFBS for multiple zinc finger transcription factors, including KLF1 and KLF3 (Fig. 4I, Table S7). CHD2 peaks that were unique to hESCs and that gained H3K27me3 in hMGEs/hcINs were also enriched for zinc finger TFBS, including motifs for KLF10 and ZEB2 (Fig. 4J, Table S7). By contrast, hMGE/hcIN-unique CHD2 peaks that co-localized with H3K27ac in hMGEs/hcINs were enriched for distinct RFX and homeodomain TFBS, including those recognized by RFX1, RFX2, and LHX2 (Fig. 4K, Table S7). These results suggest that CHD2 may play several roles in interneuron specification and differentiation, depending on its co-localization with distinct classes of transcription factors.

### CHD2 haploinsufficiency causes precocious differentiation through reduced cell cycle and increased neuronal gene expression

To further elucidate the mechanistic role of CHD2 during hcIN differentiation, we assessed how heterozygous CHD2 loss of function affected gene expression and chromatin state during hMGE specification and hcIN differentiation. For this work, we derived an hESC line with mono-allelic disruption of *CHD2* (CHD2^+/−^), modeling the *CHD2* haploinsufficiency seen in most patients with *CHD2* mutations^2,25^. The guide RNA (gRNA) used targeted the third exon of *CHD2* and created a mutant *CHD2* allele with a two base-pair frameshift, predicted to result in a premature stop codon and a severely shortened protein (88 amino acids in the mutant, versus 1,829 in the WT protein and containing no known CHD2 functional domains; Figure S6A). Western blot analysis indicated that CHD2 protein levels in the *CHD2*^+/−^ hESCs were reduced by ~ 70% (Figure S6B). As has been demonstrated by our previous work in hESCs^15^ and by others in mouse ESCs^14^, no differences in pluripotency maintenance or cell proliferation were observed in WT versus CHD2^+/−^ hESCs and both cell lines were karyotypically normal (Figure S6C).

Analyzing gene expression changes between the CHD2^+/−^ and WT models at the hMGE and hcIN stages of development showed that the majority of differentially expressed genes (DEGs) were down regulated in the CHD2^+/−^ versus WT model at both time points (Fig. 5A,B). Using our previously generated gene expression clustering (Fig. 1D, Supplemental Fig. S1A), we determined which cluster these DEGs belonged to. This analysis highlighted that, at both the hMGE and hcIN time points, DEGs that were downregulated in the CHD2^+/−^ by comparison with the WT model (CHD2^+/−^ Down) were enriched for expression clusters 4 (green), 6 (red), and 8 (orange) (Fig. 5C,D, Table S1). During differentiation, all of these clusters show decreased expression from D0 to D15 and an overall decrease in gene expression from D0 to D35 (Fig. 1D). These results indicated that CHD2 deficiency reduced the expression of genes that are normally downregulated during hMGE specification and hcIN differentiation. Enriched GO terms associated with these genes (Fig. 5E,F, Table S8) were related to early aspects of neurodevelopment or neural fate acquisition and altered cell–cell interactions (e.g. ‘Neural Tube Formation’, ‘Tissue Morphogenesis’, and ‘Tight Junction Assembly’). We also found a set of CHD2^+/−^ Down DEGs seen only at the hcIN stage; these were in expression cluster 12 (brown) (Fig. 5D); during differentiation, these genes exhibit decreased gene expression only between D15–D35, during hcIN differentiation (Fig. 1D). These genes were highly enriched for GO terms associated with cell division, such as ‘Cell Cycle Progression’ (Fig. 5G, Table S8).

At the hMGE stage, there were very few DEGs up-regulated in the CHD2^+/−^ model by comparison with WT (CHD2^+/−^ Up); however, significant numbers of CHD2^+/−^ Up genes were detected at the hcIN stage. CHD2^+/−^ Up DEGs in hcINs predominantly fell into expression clusters 1 (light blue), 2 (blue), 3 (light green), 7 (light orange), 9 (light purple), and 11 (yellow) (Fig. 5D, Table S1). During hcIN differentiation, all of these clusters exhibit increased expression at D35 versus D0. GO terms associated with these genes (Fig. 5H, Table S8) were enriched for terms related to neuronal differentiation and maturation such as ‘Synaptic Signaling’ and ‘Neuron Differentiation’.

**Figure 6:**
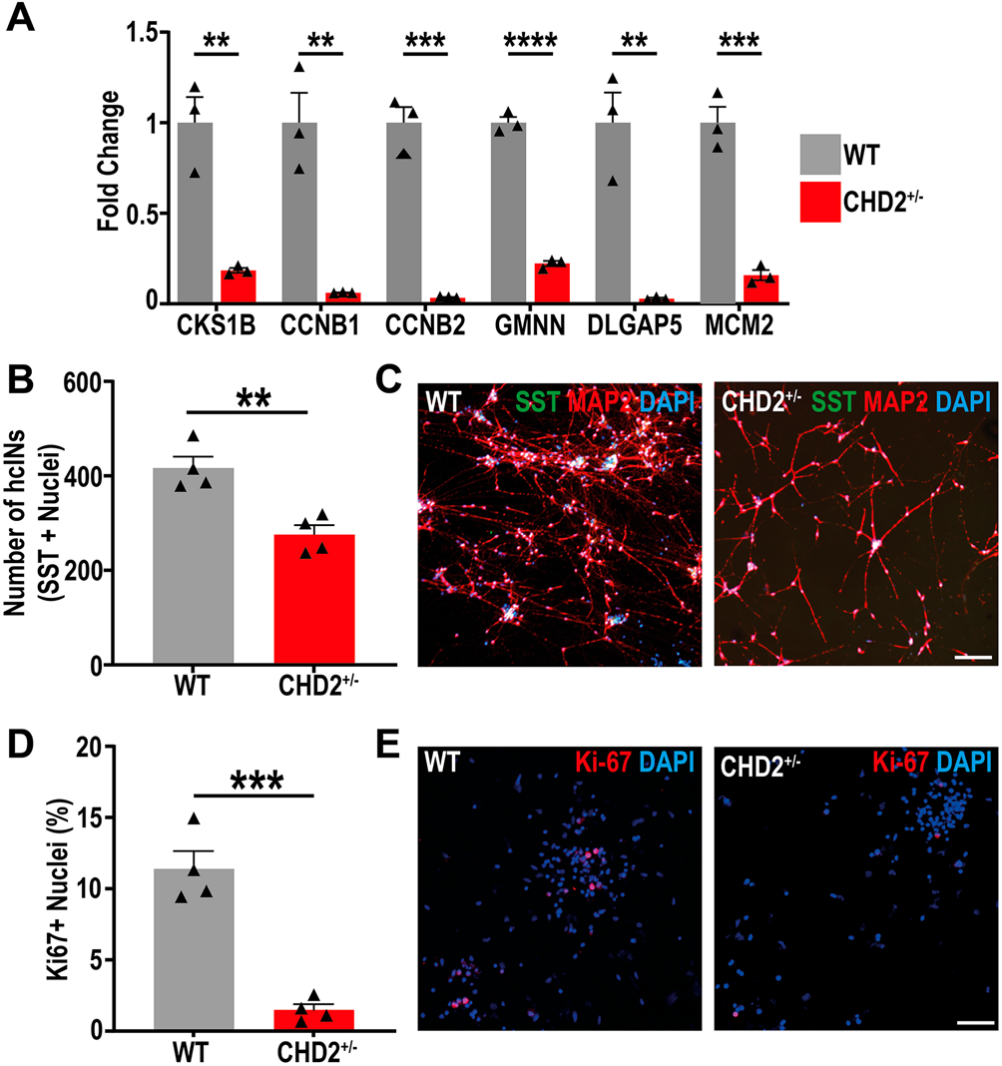
CHD2 haploinsufficiency alters the efficiency of the proliferation to differentiation transition during hcIN differentiation. (**A**) RT-qPCR was used to define the expression in WT and CHD2^+/−^ cultures at D35 of 6 significantly down-regulated DEGs (as obtained by RNA-seq analysis), that are associated with cell cycle progression. (**B**) Comparison of the numbers of SST immunopositive hcINs in WT versus CHD2^+/−^ cultures, after plating equivalent numbers of progenitors. Earlier and more efficient differentiation of progenitors resulted in a reduction in the total number of hcINs generated. n = 3 biological replicate experiments. (**C**) Representative images of SST and MAP2 staining in WT and CHD2^+/−^ D35 cultures. (**D**) The percentage of nuclei that were immunopositive for Ki-67 in the WT versus CHD2^+/−^ model at D35 of differentiation were defined. n = 3 biological replicate experiments. (**E**) Representative images of D35 cells immunostained for Ki-67. Scale bars = 100 µm. Data is represented as mean ± SEM and was analyzed by Students *t*-test: **P < 0.01 ***P < 0.001 ****P < 0.0001 versus control.

To confirm some findings from the RNA sequencing analysis conducted above, we selected six genes that were significantly down regulated in the CHD2^+/−^ model at D35 and belonged to GO terms related to cell cycle progression and we performed reverse transcription of RNA and quantitative PCR (RT-qPCR). This analysis confirmed that all six genes were significantly down regulated in the CHD2^+/−^ model, relative to wildtype (WT) cINs (Fig. 6A). To assess this phenomenon further, we examined the production of neurons in wildtype versus CHD2^+/−^ hcIN cultures by staining for Somatostatin (SST), a marker of early born hcINs. We found a significant reduction in the total number of neurons generated in CHD2^+/−^ hcIN cultures, with no change in the overall percentage of neurons that were SST immunopositive (Fig. 6B,C; Supplemental Fig. S6D). Immunostaining for NKX2.1 at day 25 also showed no significant difference in the proportion of immunopositive nuclei between WT and CHD2^+/−^ models (Supplementary Fig. S6E,F), further suggesting that CHD2 haploinsufficiency does not affect the specification of cortical interneurons.

To confirm that the loss of hcINs was a result of alteration in the numbers of mitotically cycling cells in our CHD2^+/−^ model, we performed staining for the cell proliferation marker Ki-67 at D35, finding a significant reduction in the number of Ki-67 positive cells in the CHD2 haploinsufficiency model (Fig. 6D,E). We hypothesized that the loss of proliferating cells and the corresponding reduction in the number of SST+ hcINs present in CHD2^+/−^ cultures at D35 of differentiation could result from cells prematurely exiting the cell cycle. To test this hypothesis we pulsed EdU (5-ethynyl-2′-deoxyuridine) for 2 or 12 h at D22 of differentiation and then immunostained the cells for EdU at D30. Using this metric, we found no significant differences in the number of EdU+ cells at D30 (Supplemental Fig. S6G–J). Therefore, we suggest that the reduction in the number of proliferative cells in CHD2^+/−^ cultures is unlikely to be caused by cells prematurely exciting the cell cycle, but instead reflects a more complete transition of the cell population from cycling progenitors to immature neurons by D35. Together, this analysis suggests that CHD2 deficiency may enhance the potential for hcIN differentiation.

### Epigenetic consequences of CHD2 haploinsufficiency

As CHD2 binding at both the hMGE and hcIN stages was predominantly associated with H3K37ac coenrichment, we hypothesized that the reduction of CHD2 expression could result in the loss of H3K27ac and a corresponding decrease in expression of the associated genes. To investigate this hypothesis, we defined the H3K27ac peaks in the CHD2^+/−^ model at both the hMGE and hcIN stages. We examined H3K27ac differentially bound regions (DBRs) in CHD2^+/−^ and WT model comparisons associated with genes that were also bound by CHD2 at any time point. We found no DBRs which showed increased H3K27ac in CHD2^+/−^ hMGEs compared to WT (CHD2^+/−^ Up) and very few DBRs (143 peaks) with reduced H3K27ac in CHD2^+/−^ hMGEs compared to WT (CHD2^+/−^ Down). By contrast, in hcINs we found 2308 CHD2^+/−^ Down DBRs and a smaller number of CHD2^+/−^ Up DBRs (883 peaks).

**Figure 7:**
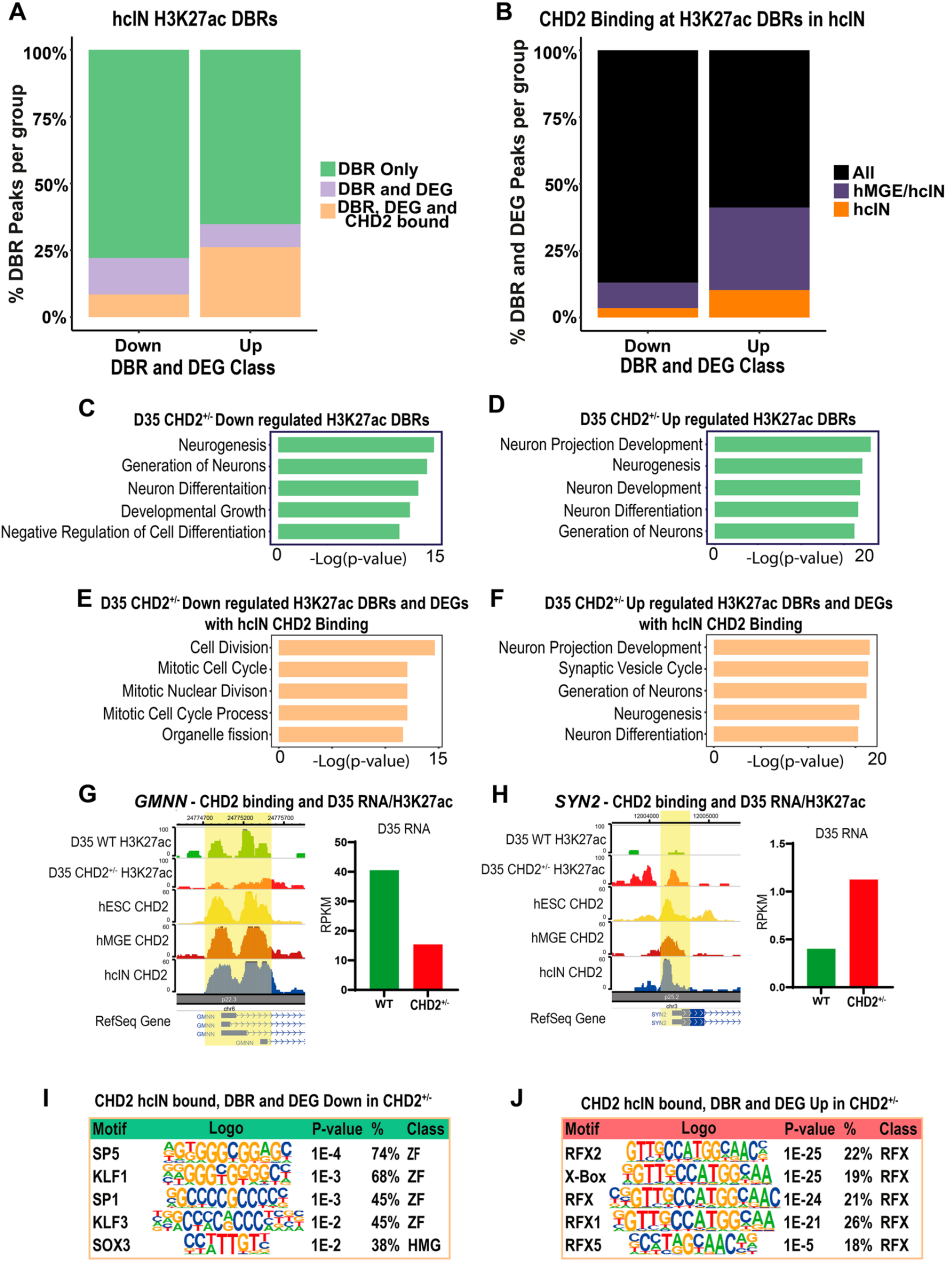
Corresponding epigenetic changes and differential gene expression resulting from CHD2 haploinsufficiency in hcINs. (**A**) Intersection of H3K27ac differentially bound regions (DBRs) and DEGs in the WT versus CHD2^+/−^ comparisons, and of CHD2 binding events in hcINs. DBRs and DEGs were required to change in the same direction in CHD2^+/−^ versus WT hcINs. (**B**) Temporal kinetics of CHD2 binding at H3K27ac DBRs associated with a DEG. The relative frequency with which DBR and DEG associated peaks were bound by CHD2 throughout differentiation (“All”) was compared was compared with the fraction of DBR- and DEG-associated peaks bound by CHD2 only at later stages of development (either at both the hMGE and hcIN stages or uniquely in hcINs). (**C**, **D**) Gene ontology (GO) enrichment analysis of DEGs associated with H3K27ac DBRs that were (**C**) down-regulated or (**D**) up-regulated in CHD2^+/−^ hcINs, by comparison with WT. Top GO terms and their -log10 p-values are shown. (**E**, **F**) Gene ontology (GO) term analysis of DEGs associated with H3K27ac DBRs that were also CHD2 bound in hcINs, where the DEGs and DBRs were either (**E**) down-regulated or (**F**) up-regulated in the CHD2^+/−^ versus WT hcINs. (**G**, **H**) Examples of H3K27ac DBRs that were associated with DEGs and with CHD2 binding in hcINs were obtained from analysis of genes associated with significant GO terms and include (**G**) *GMMN* (down-regulated DBR and DEG in the CHD2^+/−^ vs. WT comparison) or (**H**) SYN5 (up-regulated DBR and DEG in the CHD2^+/−^ vs. WT comparison). (**I**, **J**) Analysis of enriched transcription factor binding sites under DBRs that are also CHD2 bound peaks and are associated with DEGs in hcINs, with analysis considering either sequences under peaks that were (**I**) down-regulated or (**J**) up-regulated DBRs and DEGs in CHD2^+/−^ versus WT hcINs.

As there were very few H3K27ac DBRs at the hMGE stage, and the transition from proliferative hMGE progenitors to differentiated hcINs is a critical stage of hcIN development, we focused on the DBRs in hcINs. We mapped H3K27ac DBRs in hcINs to the closest TSS and quantified the number that were associated with a DEG that changed in the same direction as the DBR in the CHD2^+/−^ versus WT comparison. While most DBRs did not associate with a DEG (Fig. 7A, Table S1), 511 CHD2^+/−^ down DBR peaks were associated with a CHD2^+/−^ down DEG, while 307 CHD2^+/−^ up DBR peaks were associated with a CHD2^+/−^ up DEG. To determine which peaks could be directly affected by CHD2 deficiency, we also quantified how many of these peak-associated genes were CHD2 bound in hcINs. Surprisingly, substantially more CHD2^+/−^ up DBRs were associated with both a CHD2^+/−^ up DEG and with CHD2 binding in hcINs, relative to the same type of comparison for CHD2^+/−^ down DBRs, resulting in similar numbers of peaks in each category (195 CHD2^+/−^ down DBRs associated with a CHD2^+/−^ down DEG and with hcIN CHD2 binding, and 231 CHD2^+/−^ up DBRs associated with a CHD2^+/−^ up DEG and with hcIN CHD2 binding). Finally, to better understand the role of CHD2, we examined the temporal nature of the CHD2 binding events at these sites. CHD2^+/−^ down DBRs associated with both CHD2^+/−^ down DEGs and CHD2 binding in hcINs were largely sites where CHD2 was bound throughout development (Fig. 7B "All", Table S1). By contrast, a substantial proportion of CHD2^+/−^ up DBRs associated with a CHD2^+/−^ up DEG and with CHD2 binding in hcINs (~ 40%) were sites where CHD2 only bound at later stages of development (either at both the hMGE and hcIN stages or uniquely in hcINs, Fig. 7B, Table S1).

We went on to assess the function of DEGs that were associated with H3K27ac DBRs as described above. In these analyses, GO term enrichment was similar for both CHD2^+/−^ down and CHD2^+/−^ up data sets and predominantly featured terms related to neurogenesis and neuronal differentiation (Fig. 7C,D, Table S9). However, examining H3K27ac DBRs that were both associated with an H3K27ac DBRs and were bound by CHD2 in hcINs (Table S9) yielded enriched GO terms related to those observed in our overall DEG analysis (comparison of Figs. 5G,H, 7E,F, Table S8). CHD2^+/−^ Down DBRs associated with both a CHD2^+/−^ Down DEG and with CHD2 binding in hcINs were enriched for terms related to cell cycle progression and cell division (Fig. 7E, example in Fig. 7G, Table S9). By contrast, CHD2^+/−^ Up DBRs associated with both a CHD2^+/−^ Up DEG and with CHD2 binding in hcINs were enriched for terms related to neuronal differentiation and maturation, including genes implicated in the etiology of NDDs including autism and epilepsy (Fig. 7F, example in Fig. 7H, Table S9). These results suggest that CHD2 could play a direct role in both up-regulation of cell cycle and down-regulation of neuronal genes during hcIN differentiation, with CHD2 deficiency resulting in reduced cell cycle and increased neuronal gene expression, consistent with an alteration of differentiation timing or potential.

**Figure 8:**
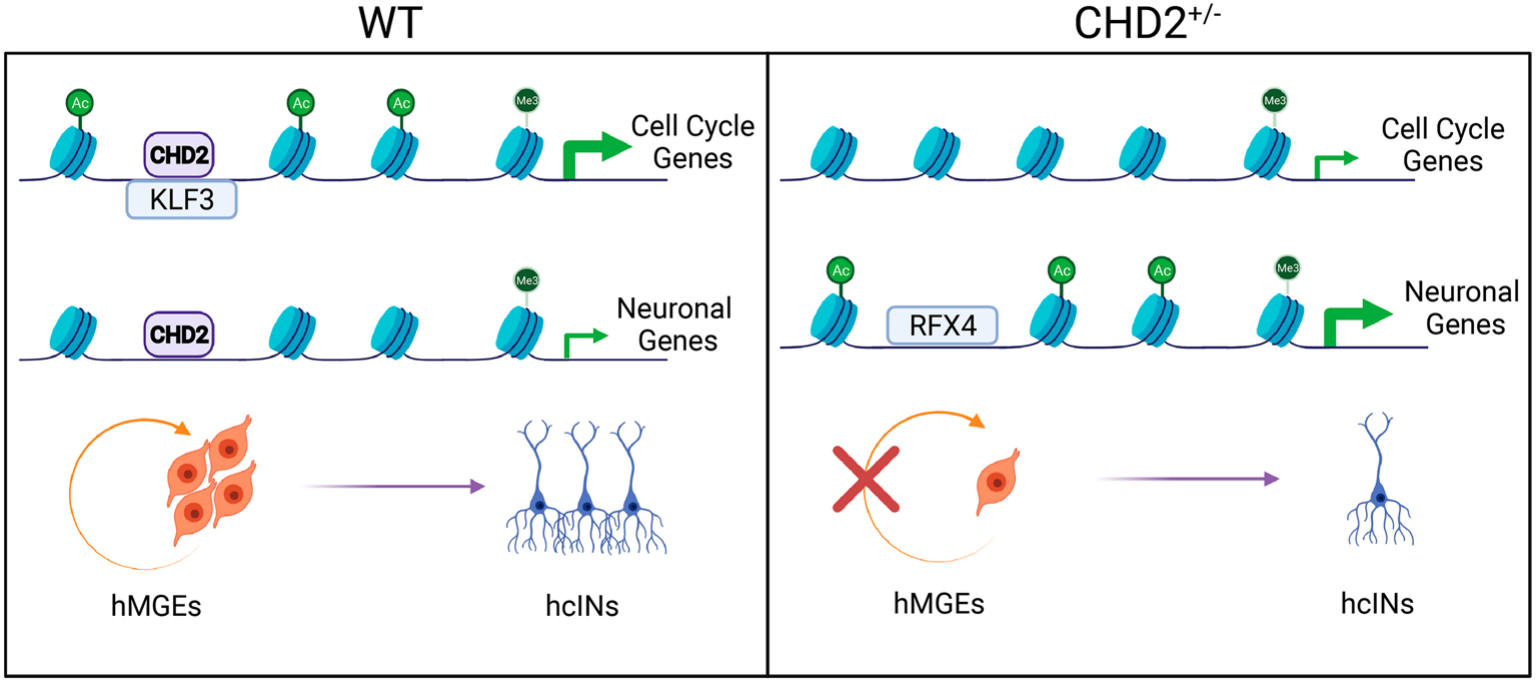
Model for CHD2 function during hcIN differentiation. During hcIN differentiation, CHD2 bound peaks exhibit high H3K27ac (Ac) and are near promoters enriched for H3K4me3 (Me) in genomic locations enriched for TFBS recognized by zinc finger TFs, including KLF3. CHD2-bound peaks with this configuration are associated with genes related to mitosis and cell division (cell cycle genes). Under conditions of CHD2 deficiency (CHD2^+/−^), H3K27ac is reduced at these sites and expression of the associated cell cycle-related genes is reduced. CHD2 binds another set of peaks without H3K27ac coenrichment in MGE progenitors. These are enriched for RFX TFBS and are associated with neuronal genes that are not highly expressed until hcIN differentiation. Under conditions of CHD2 deficiency, H3K27ac levels increase at these peaks and expression levels of these genes increase, potentially due to the presence of a chromatin environment permissive for binding of RFX TFs such as RFX4, which can promote neuronal gene expression. Ultimately, these direct requirements for CHD2 at two distinct direct target gene subsets results in more efficient differentiation in the CHD2^+/−^ model, due to consequent dysregulation of both cell cycle and neuronal genes.

Finally, we examined TF binding site enrichment under H3K27ac DBRs associated both with a DEG and with hcIN CHD2 binding. CHD2^+/−^ Down DBRs that met these criteria were enriched for zinc finger TFBS (Fig. 7I, Table S10), with the TFs that recognize these sites being most highly expressed at the hESC or hMGE stages. By contrast, CHD2^+/−^ Up DBRs were enriched for RFX factor binding motifs (Fig. 7J, Table S10), while many RFX TFs are most highly expressed during later stages of development (hMGE and hcIN). These results suggest that CHD2 could function cooperatively with zinc finger TFs to promote the expression of cell cycle genes, while it may instead antagonize the ability of RFX TFs to activate gene expression necessary for neuronal maturation. We hypothesize that these distinct activities underlie a direct requirement for CHD2-regulated target gene expression to control differentiation, and that this process is dysregulated under conditions of CHD2 deficiency (Fig. 8).

## Discussion

In this study, we obtained genome-wide binding profiles for CHD2 at multiple stages of hcIN differentiation and integrated these data with both gene expression and chromatin state data, defining roles for CHD2 in human cortical interneuron development for the first time. We also compared chromatin state and gene expression data for WT MGEs and hcINs versus those with CHD2 haploinsufficiency, to identify epigenetic mechanisms that may underlie altered regulation of gene expression resulting from CHD2 deficiency. These results provide insight into the potential consequences of pathogenic *CHD2* mutation.

We first identified CHD2 bound genomic regions in hESCs, hMGEs, and hcINs. Many regions were CHD2 bound only at one time point, reflecting stage-specific roles, while CHD2 binding was frequently associated with high expression of the corresponding gene and with key stage-specific gene functions. CHD2 binding frequently preceded increased gene expression, suggesting that it may often facilitate a permissive chromatin environment that enables other TFs to promote gene expression. We also identified distinct classes of candidate TFs that may coregulate gene expression with CHD2 at each stage of interneuron differentiation. In hESCs, CHD2 bound sites were enriched for the zinc finger TFBS ZIC3 and TEAD3, and for OCT4 and NANOG TFBS, all of which regulate pluripotency^26,27^. These findings are consistent with a prior demonstration that CHD2 and OCT4 can cooperatively regulate gene expression in mouse ESCs^14^. In hMGE progenitors, CHD2 bound locations were instead enriched for multiple homeodomain TFBS, some of which have known roles in MGE specification^28,29^. Finally, in hcINs, CHD2 bound sites were enriched both for homeodomain TFBS, some of which were also enriched at CHD2 bound sites in hMGEs, and for bHLH and RFX TFBS. Together, these findings support distinct, stage-specific roles for CHD2 during interneuron specification and differentiation and suggest TFs with which CHD2 may cooperatively regulate gene expression at each stage.

We further examined patterns of TFBS and histone modification coenrichment at CHD2 peaks. In hESCs, our results were congruent with previously published work, demonstrating CHD2 enrichment at active promoters (coenriched for H4K4me3 and H3K27ac) and enhancers (marked by H3K27ac). Similar coenrichment has been identified previously in several cell types, including hESCs, mouse ESCs, and K562 human lymphoblast cells^14,17^. CHD2 and H3K4me3 were also coenriched at hESC-specific CHD2 peaks that gained the repressive H3K27me3 modification during differentiation, after CHD2 binding was lost. These data suggest that loss of CHD2 binding is permissive for acquiring a more repressive, bivalent chromatin state and is congruent with a prior finding for mouse ESCs, which suggested that CHD2 binding could block formation of repressive chromatin^14^. During hMGE specification and hcIN differentiation, CHD2 bound sites frequently gained H3K27ac coenrichment, suggesting that this change in chromatin state may be central to CHD2-mediated target regulation during interneuron differentiation.

Assessing the effects of CHD2 haploinsufficiency provided additional information regarding its roles in interneuron differentiation. A particularly interesting gene set was down-regulated in CHD2^+/−^ hcINs, relative to WT conditions, and included many cell cycle genes, the expression of which normally decreases in the WT model only after MGE specification and during hcIN differentiation. By contrast, genes upregulated in CHD2^+/−^ hcINs were largely related to neuronal differentiation and function. Together, these data support a role for CHD2 in controlling the transition from proliferative hMGE progenitor to post-mitotic hcIN. These results are reminiscent of findings from prior murine and human studies of CHD2 loss, which resulted in reduced progenitor proliferation and accelerated differentiation^16,19,30^. For example, Shen et al.^16^ found premature neuron differentiation upon shRNA-based *Chd2* knockdown in mouse cortex. Kim et al.^19^ also identified reduced cIN progenitor numbers in the MGE of *Chd2*^+/−^ mice, resulting in abnormal transcriptomic and electrophysiological properties of mature cINs. During differentiation of the LUHMES neural progenitor cell line, Lalli et al.^30^ also showed that *CHD2* knockdown reduced progenitor proliferation. Finally our previous work on a CHD2 knock-out hESC model showed a loss of TUBB3+ cells and reduced expression of both hMGE (LHX6 and DLX2) and mature hcIN (GAD2) markers^15^. These results are also consistent with our hypothesis that CHD2 functions to regulate the transition between mitotically cycling progenitors and maturing hcINs. However, the prior findings additionally suggested that, either directly or indirectly, the complete loss of CHD2 resulted in functional neuronal alterations, including a significantly lower threshold for action potential firing and larger peak sodium currents^15^.

While prior studies are compatible with our findings here, supporting a role for CHD2 in regulating the transition of cortical neurons from proliferation to differentiation, the mechanistic basis of this requirement was previously unknown. Based upon our data, we propose a model for CHD2 function during hcIN differentiation (Fig. 8). Our data indicates that CHD2 directly regulates two broad classes of genes, those involved in maintaining hMGE progenitors as proliferative cells (cell cycle genes, Fig. 8) as well as those that promote hcIN differentiation (neuronal genes, Fig. 8). CHD2 bound sites at cell cycle genes coenrich for H3K27ac and are associated with genes that are highly expressed in hMGE progenitors, relative to hcINs. CHD2 deficiency causes H3K27ac to be lost at these sites, while expression of the associated genes decreases. We hypothesize that CHD2 deficiency reduces the active, open chromatin state (marked by H3K27ac) at these sites, reducing the ability of TFs to bind and activate cell cycle gene expression (Fig. 8). Conversely, at CHD2 bound neuronal genes, CHD2 deficiency results in H3K27ac gain at CHD2 peak locations and a corresponding increase in expression of the associated neuronal gene. We hypothesize that CHD2 binding to peaks associated with these neuronal genes may instead antagonize the recruitment of TFs such as RFX4, which can activate neuronal gene expression (Fig. 8); therefore, reducing CHD2 levels may indirectly promote neuronal gene expression by facilitating transactivation of regulatory elements by RFX or other TFs. Together, these data support direct CHD2 requirements at two distinct classes of target genes to regulate cell cycle and neuronal gene expression, respectively, during hcIN differentiation. Further work to test the predictions of this model, including determining how CHD2 haploinsufficeny or pathogenic mutation alters its genome binding profile and, consequently, how this alters TF binding and activity at CHD2 peak locations, will provide further insight into the mechanisms by which this regulation occurs.

These results also suggest altered mechanisms by which *CHD2* mutations could cause neurodevelopmental phenotypes. For example, reduced expression of genes that promote neural progenitor proliferation under CHD2 deficient conditions could contribute to the microcephaly seen in ~ 20% of patients with *CHD2* mutations^2^. This dysregulation could also reduce interneuron numbers, disrupting the balance between cortical excitatory and inhibitory neuronal activity to contribute to epileptic encephalopathy^31^. Likewise, many neuronal genes with H3K27ac gain and increased expression under CHD2 deficient conditions are linked to the etiology of autism and other NDDs, including *MYT1L*, *NRXN3*, *SLC12A5*, and *SNAP25* (Supplemental Table S9)^32–35^. Our finding here suggesting that CHD2 could potentially antagonize the binding or activity of the RFX TFs is also particularly interesting, as recent work has linked mutations in *RFX* genes to neurodevelopmental disorders and demonstrated that TFBS recognized by the RFX TFs are enriched in cis-regulatory regions of ASD risk genes^36^. Together, these data describe a network of direct CHD2-dependent target genes implicated in NDDs, the regulation of which is likely to be disrupted by many *CHD2* pathogenic mutations. Given the link between *CHD2* pathogenic mutation and ASD, our findings here provide a set of candidate cis-regulatory elements under the direct control of CHD2 that can be used to further investigate functional interplay between CHD2 and the RFX TFs in regulating neuronal gene expression and how its disruption could contribute to the etiology of ASD and other neurodevelopmental disorders.

Finally, while CHD2 deficiency may impact hcINs most significantly^15,16^, it will also be important to further assess its role in human cortical excitatory neurons, as these play an equally important role in properly balancing neuronal excitation and inhibition. Future work to examine how pathogenic mutations in different CHD2 functional domains alter chromatin binding and activity would contribute to our understanding of mechanisms of CHD2-dependent gene regulation during neurodevelopment and their perturbation in patients with *CHD2* mutations. Individuals with *CHD2* mutation exhibit a range of phenotypes and clinical diagnoses, including epilepsy, ASD, or both, as well as varying severity of clinical phenotypes. While this may make linking mechanistic consequences of different classes of *CHD2* mutations to clinical phenotypes challenging, such cross-comparisons could be very informative. We hypothesize that CHD2's stage-specific roles, suggested by our work here, and the potential for *CHD2* mutations to differentially affect neurodevelopmental gene regulation could contribute to the heterogeneity of patient diagnoses, through varying effects on the ability of CHD2 to bind target subsets or to interact with TFs. This could affect the timing of progenitor proliferation and differentiation in some cases, while additionally or instead altering control of gene expression required for neuronal maturation or function in others. The data obtained here regarding CHD2's direct targets, contextual information related to how CHD2 regulates these targets, and mechanisms by which its deficiency affects hcIN development provides a foundation for understanding neurodevelopmental gene dysregulation that may underlie patient phenotypes. These findings could ultimately be used to guide the development of targeted therapies that focus on restoring chronically perturbed pathways and processes, with the potential to ameliorate clinical symptoms.

## Methods

### Cell culture and differentiation

Work with hESCs was performed in approval and accordance with our institutional Embryonic Stem Cell Research Oversight Committee (ESCRO) under protocol #12-002. All hESCs were maintained under feeder-free conditions on Matrigel (Corning) in mTeSR1 (STEMCELL Technologies) and all experiments were carried out in H9 hESCs, which were obtained from WiCell Research Institute. Specification of hESCs as MGE progenitors (hMGEs) and differentiation into hcINs was performed using our previously described protocol^15^. Briefly, EBs were produced either in V-bottom 96-well non-adherent plates or using AggreWell™800 Microwell Culture Plates (STEMCELL Technologies). hESCs were dissociated with Accutase (Life Technologies) and 40,000 cells per well were added to V-bottom 96-well non-adherent plates (Corning) or 1.5 × 10^6^ cells per well on an AggreWell™800 Microwell Culture Plate. For AggreWell™800 Microwell Culture Plates, EBs were formed in EB formation media (STEMCELL Technologies) for 3 days before differentiation was started (day (D) 0) by switching to hcIN differentiation media. For V-bottom plates, EBs were formed in hcIN differentiation media, which constituted day 0 of differentiation. hcIN differentiation media contained Neurobasal-A (Life Technologies), B-27 supplement (without Vitamin A; Life Technologies), 10 µM SB-431542 (Tocris Biosciences), 100 nM LDN-193189 (Tocris Biosciences), 1 µM Purmorphamine (Calbiochem), and 2 µM XAV-939 (Tocris Biosciences). For the first 8 days of differentiation, media also contained 10 µM Y-27632 (Tocris Biosciences). EBs made in V-bottom plates were maintained for 4 days before moving to an orbital shaker set to 80 rpm, whereas EBs generated using AggreWell™800 Microwell Culture Plates were maintained on an orbital shaker from D0 of differentiation. On D10 of differentiation, EBs were plated onto Matrigel- and laminin- (5 µg/mL; Sigma) coated plates. At D15 of differentiation, cells were considered hMGE progenitors and were maintained in hcIN differentiation media supplemented with SB-431542, LDN-193189, Purmorphamine, and XAV-939. For terminal differentiation neurospheres were formed from hMGE progenitors using either V-bottom plates or AggreWell™800 Microwell Culture Plates as previously described, in hcIN differentiation media supplemented with 1 µM Purmorphamine. Neurospheres were maintained for 2 days in the required plates and then were maintained on an orbital shaker for 2 days at 80 rpm. After 20 days of differentiation, neurospheres were plated onto Matrigel- and laminin- (5 µg/mL) coated plates or chamber slides and maintained in hcIN differentiation media supplemented with 1 µM Purmorphamine. From day 27–31, DAPT (10 µM; Tocris Biosciences), ascorbic acid (200 µM; Sigma), and BDNF (20 ng/mL; PeproTech) were added to the media, and from day 31–35, DAPT was replaced with cAMP (200 µM; Sigma). Cells were harvested at days 0 (hESCs), 15 (hMGEs) and 35 (hcINs) for assays, including RNA-sequencing (RNA-seq), RT-qPCR, ChIP-seq, and CUT&Tag. On D0 and D15, hESCs and hMGEs, respectively, were harvested by dissociation with Accutase, and on day 35, Neural Rosette Selection reagent (STEMCELL Technologies) was used to isolate purified populations of differentiated neurons and remove remaining undifferentiated cells from these cultures, followed by dissociation of the remaining cells with Accutase, and plating as neuronal monolayers for further culture.

### Modeling CHD2 haploinsufficiency

To model CHD2 haploinsufficiency, a heterozygous mutant (CHD2^+/−^) hESC line was constructed by CRISPR-based gene disruption in wild type (WT) H9 hESCs, using our previously described approach^15^. Heterozygous *CHD2* mutation was validated by Sanger sequencing and all lines were shown to be karyotypically normal, with testing conducted by the Washington University School of Medicine Cytogenetics and Molecular Pathology Laboratory.

### CUT&Tag

CUT&Tag was performed on WT hESCs (day 0), hMGEs (day 15), and hcINs (day 35) for CHD2 and H3K27me3. The protocols.io V.2. protocol^37^ was followed, using a minimum of 200,000 cells per sample. The protein A-Tn5 fusion protein (pA-Tn5) was provided by the laboratory of Steve Henikoff and the antibodies used were Rat anti-CHD2 (Sigma MABE873) and Rabbit anti-H3K27me3 (Cell Signaling Technology #9733). Secondary antibodies used were Rabbit anti-Rat IgG (abcam ab6703) and Guinea Pig anti-Rat IgG (Antibodies-Online ABIN101961). Samples with unique dual-end indexes were pooled in equimolar concentrations and were sequenced by the Washington University Genome Technology Access Center (GTAC) @MGI on the NovaSeq-6000 sequencer with the S4 flow cell as 2 × 150 bp paired-end reads at a depth of at least 10 million (M) reads per sample. Raw reads from CUT&Tag samples were processed by AIAP (v1.1) using human genome hg38 as a reference to perform read quality control, alignment, quantification, and peak calling^38^. Peaks from replicates were intersected using bedtools intersect peak with options -f 0.25 -F 0.25 -e. Peaks identified as intersecting in 2 or more out of 4 total replicates were defined as reproducible peaks.

### ChIP-seq

ChIP-seq was performed on WT hESCs (day 0), hMGEs (day 15), and hcINs (day 35) for H3K27ac and H3K4me3 and on the line with heterozygous disruption of CHD2 (CHD2^+/−^) in hMGEs (day 15), and in hcINs (day 35) for H3K27ac, as described previously^15^. The antibodies used were Rabbit anti-H3K27ac (Millipore 07-360) and Rabbit anti-H3K4me3 (Millipore 07-473). Library preparation and sequencing were performed by GTAC@MGI using the HiSeq3000 sequencer to obtain at least 30 M single end 50 base pair reads per sample. ChIP-seq data analysis was performed as described previously^15^.

### RNA-seq

For comparison of gene expression in WT and CHD2^+/−^ cells during hcIN differentiation, RNA was collected from hMGEs (day 15) and hcINs (day 35) from 4 biological replicate experiments per sample type, with WT and CHD2^+/−^ cells differentiated in parallel to generate each biological replicate. RNA was collected and quantified as previously described^39^. RNA-seq library preparation and Illumina sequencing was performed by the GTAC using the SMARTer Ultra Low RNA kit (Takara-Clontech) per the manufacturer’s instructions. Paired-end 150 bp reads were obtained using an Illumina NovaSeq-6000 sequencer with the S4 flow cell, to a depth of ~ 30 M reads per sample. The transcriptomic data and epigenomic data obtained here was also compared with datasets obtained in our previous work^15^ that describes transcriptomic changes accompanying differentiation from hESCs (day 0), to hMGE progenitors (day 15), and during hcIN maturation (day 35).

Raw reads from RNA-seq samples were quality trimmed using cutadapt (v2.4) with options—quality-cutoff = 15,10—minimum-length = 36^40^. Reads were aligned using STAR (v2.5.4b) using human genome hg38 as a reference with Gencode V27 annotations^41^. Reads were quantified at the gene level using featureCounts (v1.6.4) from the subread package^42^. For differential expression analysis, gene-level quantification from featureCounts were normalized to counts per million (CPM) using the CPM function in edgeR in R (v3.6)^43^. Genes with expression under 1 CPM in more than half of the samples compared were excluded from analysis. Differential expression analysis was performed with DESeq2 in negative binomial mode using counts for genes that passed the 1 CPM cutoff^44^. A twofold expression change, expression higher than 1 CPM, and a Benjamini and Hochberg false discovery rate (adjusted *p* value) of < 0.05 were set as cutoff values for a gene to be considered differentially expressed.

### Quantitative PCR

RNA was isolated using the NucleoSpin RNA kit (Macherey–Nagel), was quantified using a NanoDrop ND-1000 spectrophotometer (Thermo Scientific) and 1000 ng was used to make cDNA using the High-Capacity cDNA Reverse Transcription Kit (Applied Biosystems). Equal quantities of cDNA were used as template for the qPCR using the Applied Biosystem StepOne Plus quantitative PCR system. GAPDH was used as an endogenous quantitative control and all primers used can be found in Table S11. Data were generated from 4 biological replicate samples, each of which consisted of 3 technical replicates. All statistics were performed by calclating the relative abundance, and data is presented as fold change by comparison with WT.

### Annotation and motif analysis of peak sets

Peak sets were annotated using the annotatePeaks.pl function in HOMER2 using human reference hg38 and GENCODE V27 annotation^45^. Promoter regions were defined as +/- 2kb from the transcription start site. Genes and genomic region annotations associated with peaks were defined using calls based on the nearest transcription start site (TSS) by HOMER. Motif analysis was performed using the findMotifsGenome.pl function within HOMER2 with hg38 as reference and option -size given.

### Data clustering and visualization

Clustering analysis was performed using WT gene expression data from hESCs (day 0), hMGEs (day 15), and hcINs (day 35). All genes with a fold change of less than 1.5 from hESCs to hMGEs or from hMGEs to hcIN were eliminated from analysis. Clustering was performed on gene expression (RPKM) values using the kmeans function in the R statistical package^46^, with a K value of 12 determined using the elbow method of the sum of squared errors. To generate deepTools plots, representative signal density bigwig files chosen at random from the replicates and normalized to a depth of 10 M reads by AIAP were used for visualizations with deepTools (v3.5.0)^47^. The deepTools signal matrix was generated using selected peak sets by the computeMatrix function in reference-point mode with options -a 2500 -b 2500 -bs 50—missingDataAsZero—referencePoint center. Plots were generated using the plotHeatmap function with options -zMin 0 -zMax 60 -yMin 0 -yMax 60 for uniform signal density scaling. Heatmaps were created using ClustVis^48^, with rows centered and unit variance scaling applied to rows. Rows were clustered using correlation distance and average linkage. Gene ontology enrichment analysis was done using the ToppFun tool from the ToppGene suite^49^ and reporting FDR corrected p-values generated using the probability density function methodology. Gene network analysis was done using the CompBio tool from the PercayAI platform^50^, using the 1000 genes with CHD2 binding closest to the TSS (500 genes upstream and 500 downstream) as input to assess each CHD2 bound gene set. Further network analysis and visualization was done using ClueGO (Cytoscape)^51^.

### Immunofluorescence and Edu staining

Where appropriate, cells were treated with 5 µM Edu for 2 or 12 h at day 20 of differentiation. Cells were fixed in 4% Paraformaldehyde and those that were incubated with Edu were stained using the Click-iT™ EdU Cell Proliferation Kit for Imaging and Alexa Fluor™ 555 dye (Thermo Fisher Scientific) as per the manufacturers instructions, after which samples were treated the same as those that were not incubated with Edu. For immunocytochemistry, cells were blocked using 5% donkey serum and 0.1% Tween20 (Sigma). Primary antibodies were: mouse anti-MAP2 (Millipore Sigma, M1406), rabbit anti-SST (Peninsula Labs, T-4102), rabbit anti-TTF1 (abcam, ab204411), and rabbit anti-KI67 (abcam, ab16667) and were added overnight at 4 °C. Cells were washed and incubated with secondary antibodies (donkey anti-mouse 555 and donkey anti-rabbit 488 (ThermoFisher Scientific A-31570 and A-21206, respectively) for 1 h at room temperature. Cells were counterstained using DAPI (1 µg/mL) and mounted using ProLong™ Glass Antifade Mountant (ThermoFisher Scientific). Images were obtained using a spinning-disk confocal microscope (Quorum), using MetaMorph software and image processing with ImageJ. Quantification was performed using a minimum of 3 random images (technical replicates) taken from a minimum of 3 independent biological replicates. Quantification was performed using CellProfiler, requiring a minimum of 80% overlap between immunostaining and DAPI staining for positivity to be registered.

### Western blotting

Protein from WT and CHD2^+/−^ embryonic stem cells was isolated in RIPA buffer and western blotted to detect CHD2 protein using the Rat anti-CHD2 (Sigma MABE873) antibody. Visualization was performed by chemiluminescence, CHD2 protein was detected at its predicted molecular weight of 200 kDa, and CHD2 band intensity was normalized to the GAPDH loading control, detected on the same blot.

### Statistical analysis

Where appropriate, statistical analysis was carried out using a combination of GraphPad Prism version 9 (GraphPad Software; La Jolla, CA, USA, available from www.graphpad.com) and RStudio version 3.5.1 (RStudio: Integrated development environment for R; Boston, MA, USA. Available from www.rstudio.org). All technical replicates were averaged before statistical analysis and specific statistical tests used for each data analysis are detailed in the figure legends. The number of separate differentiations used for each analysis is described in the figure legends as n.

## Supplementary Information

Supplementary Information 1.

Supplementary Information 2.

Supplementary Information 3.

Supplementary Information 4.

Supplementary Information 5.

Supplementary Information 6.

Supplementary Information 7.

Supplementary Information 8.

Supplementary Information 9.

Supplementary Information 10.

Supplementary Information 11.

Supplementary Information 12.

Supplementary Information 13.

Supplementary Information 14.

Supplementary Information 15.

Supplementary Information 16.

Supplementary Information 17.

Supplementary Information 18.

## Data Availability

All data generated and analyzed during this study are included in this published article, while original unprocessed data is publicly available through the Gene Expression Omnibus (GEO) repository (Superseries GSE182784). The CHD2^+/−^ hESC line used in this study is available from the investigator by material transfer agreement with Washington University.
