# Supplementary figures and images for: Regulation of human cortical interneuron development by the chromatin remodeling protein CHD2
Source: Sci Rep. 2022 Sep 17;12:15636. doi: 10.1038/s41598-022-19654-y (PMC9482661; doi:10.1038/s41598-022-19654-y)

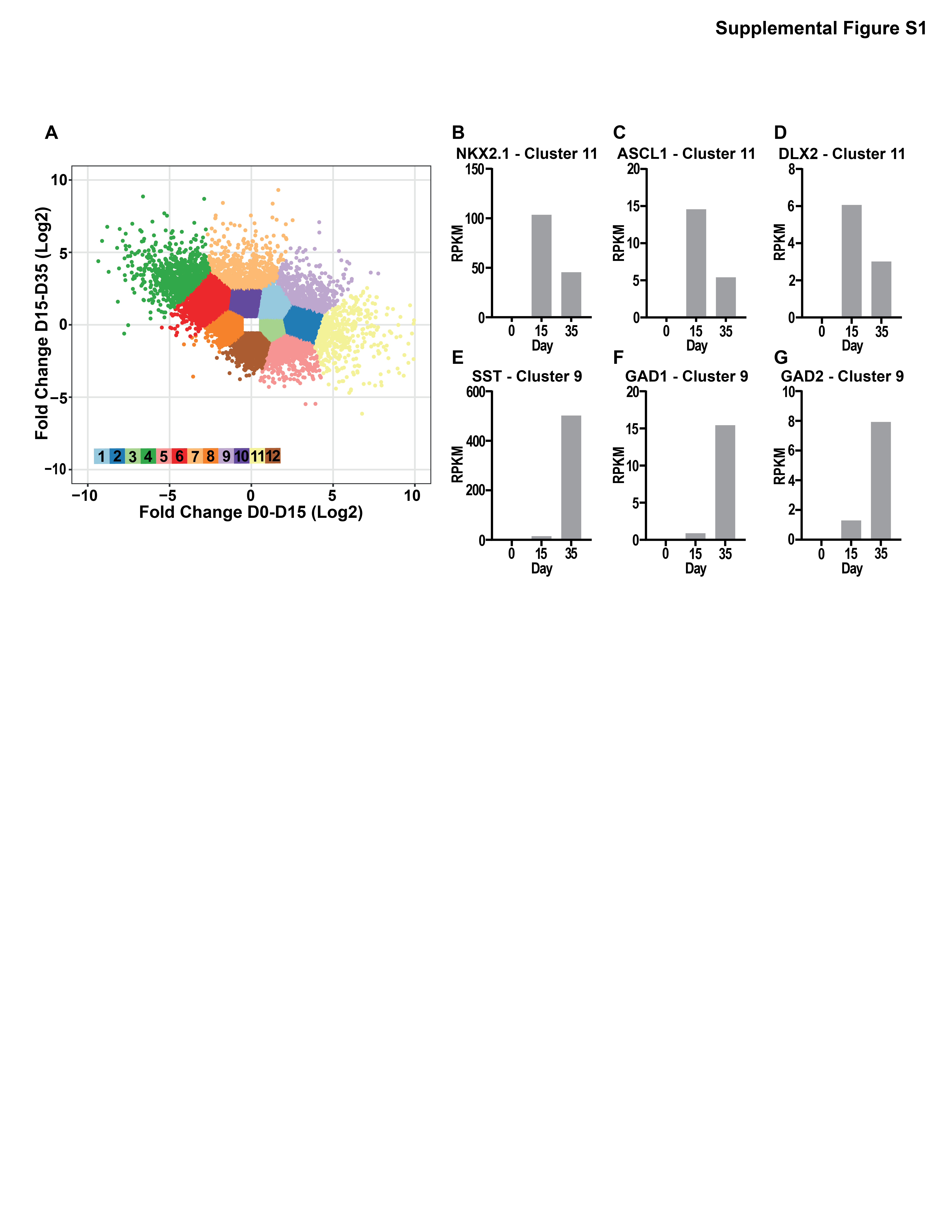

Supplement: Supplementary file 12 — Supplementary Information 12. [file 41598_2022_19654_MOESM12_ESM.jpg]

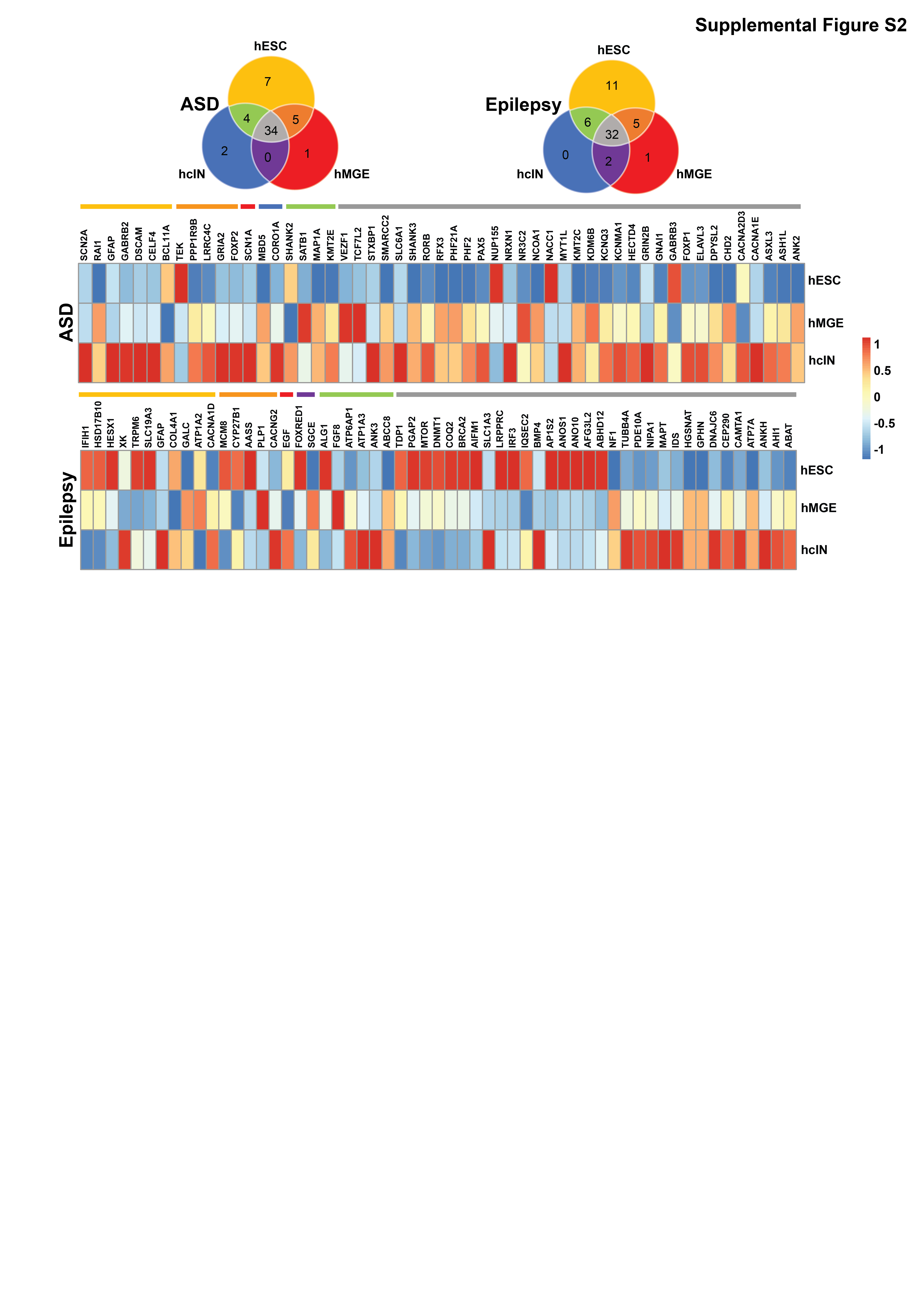

Supplement: Supplementary file 13 — Supplementary Information 13. [file 41598_2022_19654_MOESM13_ESM.jpg]

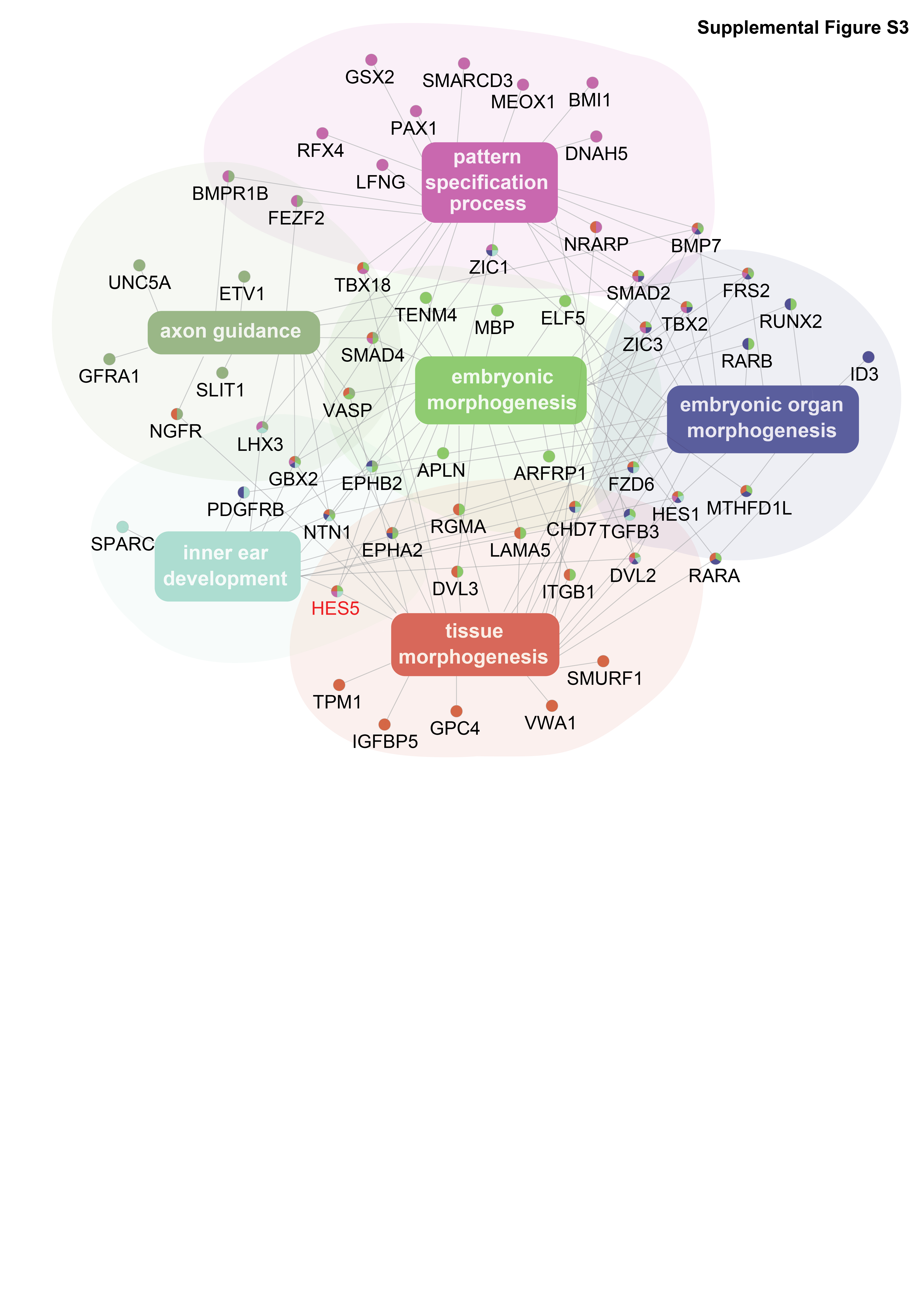

Supplement: Supplementary file 14 — Supplementary Information 14. [file 41598_2022_19654_MOESM14_ESM.jpg]

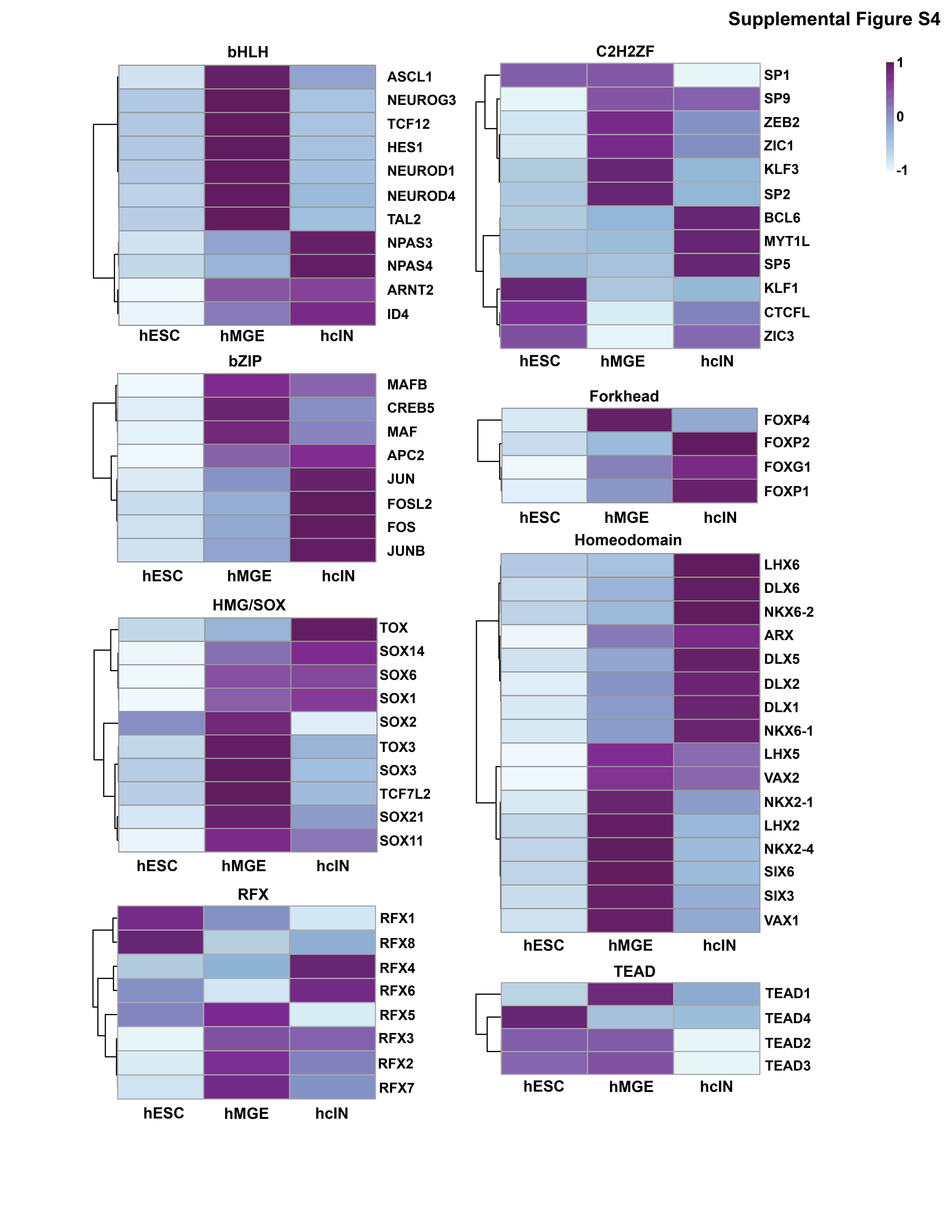

Supplement: Supplementary file 15 — Supplementary Information 15. [file 41598_2022_19654_MOESM15_ESM.jpg]

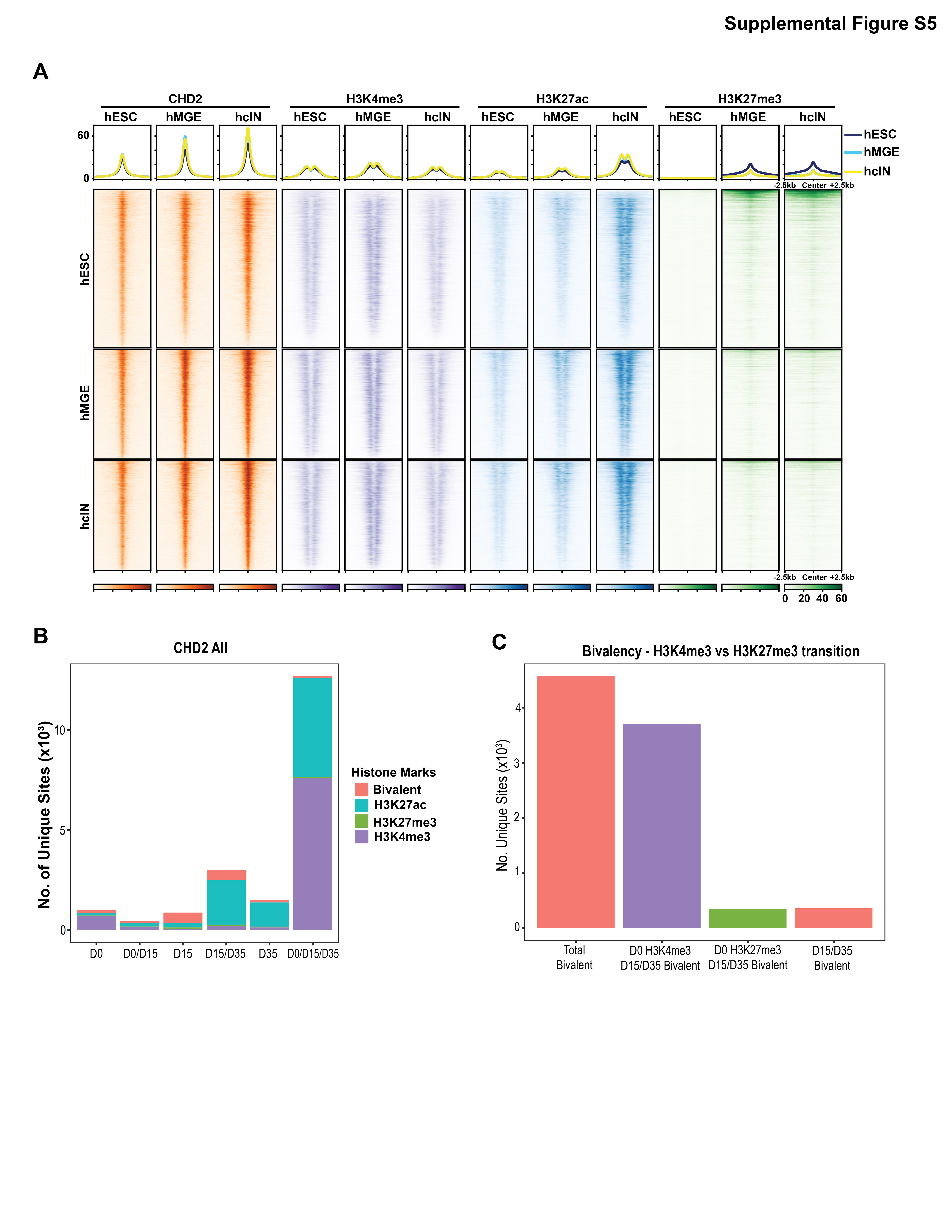

Supplement: Supplementary file 16 — Supplementary Information 16. [file 41598_2022_19654_MOESM16_ESM.jpg]

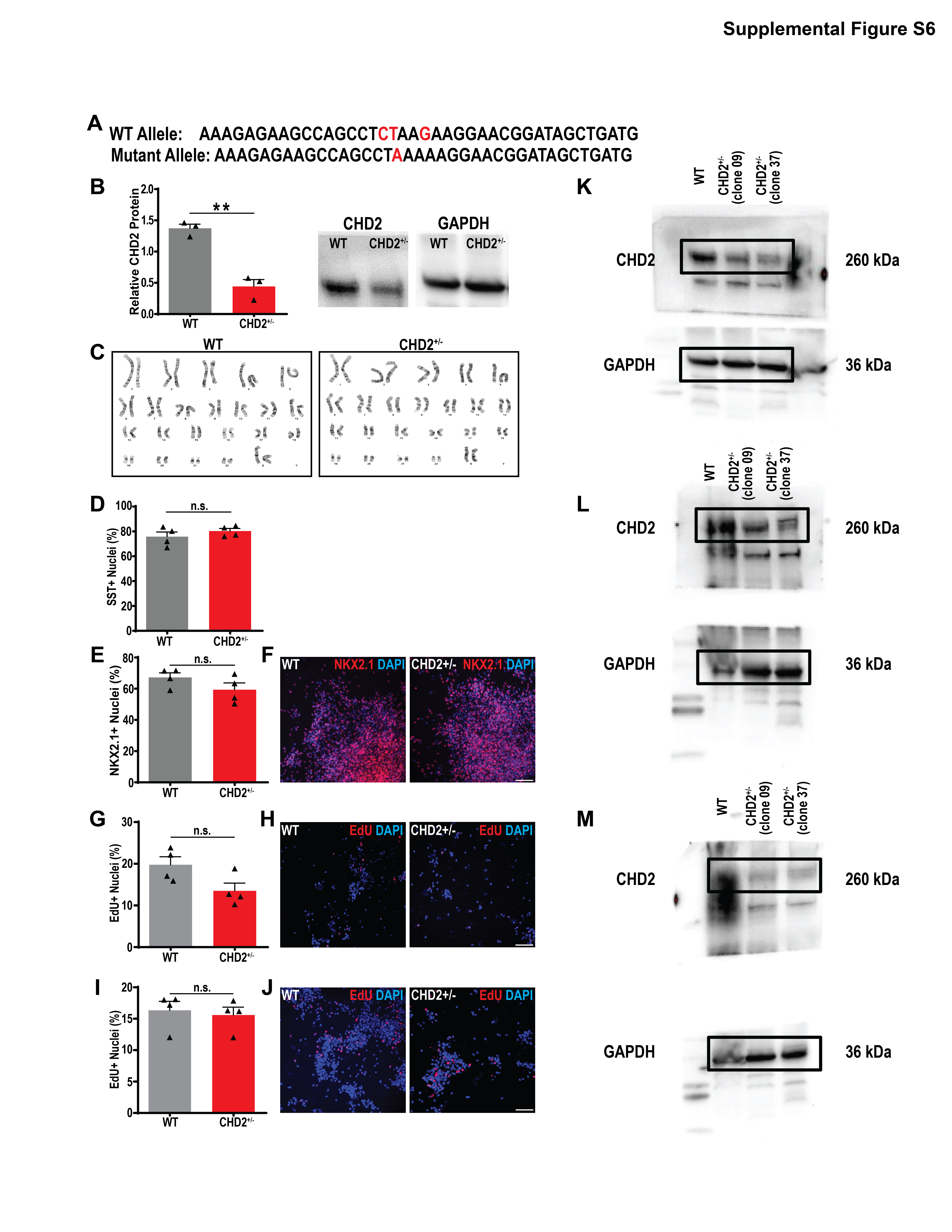

Supplement: Supplementary file 17 — Supplementary Information 17. [file 41598_2022_19654_MOESM17_ESM.jpg]
