# Supplementary material for: Regulation of human cortical interneuron development by the chromatin remodeling protein CHD2
Source: Sci Rep. 2022 Sep 17;12:15636. doi: 10.1038/s41598-022-19654-y (PMC9482661; doi:10.1038/s41598-022-19654-y)
Supplement: Supplementary file 18 — Supplementary Information 18. [file 41598_2022_19654_MOESM18_ESM.docx]

**Supplemental Table Legends**

**Supplemental Table S1. Master sheet integrating all types of data used in this paper for all CHD2 peaks.** All hg38 peaks bound by CHD2: at any stage (in day (D) 0 hESCs, D15 hMGE progenitors, or D35 hcINs 'all'), in a stage-specific manner ('unique'), or at two or more stages (for example, D0_D15pk indicates a peak bound by CHD2 in hESCs and hMGEs) are indicated (columns A-K). These were integrated with peaks where >25% of the CHD2 peak sequence intersected with a histone modification peak for H3K27ac, H3K4me3, or H3K27me3 at all three stages during differentiation of wild type (WT) hESCs into hcINs (columns L-T). These peaks were also intersected in the same manner with peaks that were differentially acetylated (H3K27ac differentially bound regions-DBRs) in WT versus CHD2^+/-^ hMGEs (D15) or hcINs (D35)(columns U-X). Peaks were annotated to the nearest transcription start site and differentially expressed genes (DEGs) in the WT versus CHD2^+/-^ RNA-seq data comparison are also indicated (columns Y-AE). Expression during differentiation of WT hESCs into hcINs (average RPKM values across replicates) and differential expression cluster numbers are also shown (columns AF-AI).

**Supplemental Table S2. Clustering analysis of differential gene expression during hcIN differentiation of wild type hESCs.** Clustering analysis was performed using WT gene expression data from hESCs (day (D) 0), hMGEs (D15), and hcINs (D35). All genes with a fold change of less than 1.5 from hESCs to hMGEs or from hMGEs to hcIN were eliminated from analysis. Clustering was performed on gene expression (RPKM) values using the kmeans function in the R statistical package^1^ to model 12 expression clusters.

**Supplemental Table S3. Enriched gene ontology (GO) terms for each set of CHD2 peak-associated genes.** All significant terms related to enriched biological processes, molecular functions, cellular components, and disease are provided for each CHD peak-associated gene set.

**Supplemental Table S4. Transcription factor binding site (TFBS) motifs enriched under each CHD-bound peak set.** All enriched motifs predicted for each set of stage-specific 'unique' peaks is shown, with the p-value and enrichment of each TFBS in target peaks versus background used to select the most highly enriched motifs.

**Supplemental Table S5. Expression of human transcription factors during hcIN differentiation.** RNA-seq data for hESC-hcIN differentiation was collected for all human transcription factors ^2^ and was used to define the expression of TFs in the classes with enriched TFBS under CHD2 peaks. Average RPKM values are shown.

**Supplemental Table S6. Enriched gene ontology (GO) terms for genes associated with each set of CHD2 and histone modification coenriched peaks.** Top GO terms were defined for: **(A)** CHD2 and H3K27ac coenriched peaks in hESCs, **(B)** CHD2 and H3K4me3 coenriched peaks in hESCs, **(C)** CHD2 and H3K27me3 coenriched peaks in hMGEs and hcINs, **(D)** CHD2 and H3K27ac coenriched peaks in hMGEs and hcINs.

**Supplemental Table S7. Transcription factor binding site (TFBS) motifs enriched under each set of CHD2 and histone modification coenriched peaks.** All enriched motifs predicted for each set of coenriched peaks is shown, with the p-value and enrichment of each TFBS in target peaks versus background used to select the most highly enriched motifs for: **(A)** CHD2 and H3K27ac coenriched peaks in hESCs, **(B)** CHD2 and H3K4me3 coenriched peaks in hESCs, **(C)** CHD2 and H3K27me3 coenriched peaks in hMGEs and hcINs, **(D)** CHD2 and H3K27ac coenriched peaks in hMGEs and hcINs.

**Supplemental Table S8. Enriched gene ontology (GO) terms for subsets of differentially expressed genes in wildtype (WT) versus CHD2^+/-^ hMGEs and hcINs.** Top GO terms were defined for: **(A)** Genes down-regulated in CHD2^+/-^ versus WT hMGE progenitors and in expression clusters 4, 6, 8, and 10, **(B)** Genes down-regulated in CHD2^+/-^ versus WT hcINs and in expression clusters 4, 6, 8, and 10, **(C)** Genes down-regulated in CHD2^+/-^ versus WT hcINs and in expression cluster 12, and **(D)** Genes up-regulated in CHD2^+/-^ versus WT hcINs and in expression clusters 1-3, 7, 9 and 11. **(E-F)** DESeq2 output for the day 15 **(E)** and day 35 **(F)** RNA-seq analysis, showing the log2 fold changes and adjusted p-values.

**Supplemental Table S9. Enriched gene ontology (GO) terms for subsets of genes associated with differential H3K27ac in wildtype (WT) versus CHD2^+/-^ hMGEs and hcINs (differentially bound regions or DBRs).** Top GO terms were defined for genes associated with: **(A)** reduced H3K27ac in CHD2^+/-^ versus WT hcINs, **(B)** increased H3K27ac in CHD2^+/-^ versus WT hcINs, **(C)** reduced H3K27ac and reduced expression (differentially expressed gene; DEG) in CHD2^+/-^ versus WT hcINs and with CHD2 binding in hcINs under WT conditions, and **(D)** increased H3K27ac and increased expression (differentially expressed gene; DEG) in CHD2^+/-^ versus WT hcINs and with CHD2 binding in hcINs under WT conditions. **(E)** Neurodevelopmental disorder-related genes with increased H3K27ac and increased expression (differentially expressed gene; DEG) in CHD2^+/-^ versus WT hcINs and with CHD2 binding in hcINs under WT conditions.

**Supplemental Table S10. Transcription factor binding site (TFBS) motifs enriched under each set of CHD2 and histone modification coenriched peaks.** All enriched motifs predicted for each set of coenriched peaks are shown, with the p-value and enrichment of each TFBS in target peaks versus background used to select the most highly enriched motifs for: **(A)** peaks bound by CHD2 in hcINs that also exhibit reduced H3K27ac (DBRs) in CHD2^+/-^ versus WT hcINs and that are also associated with a DEG with reduced expression in CHD2^+/-^ versus WT hcINs. **(B)** peaks bound by CHD2 in hcINs that also exhibit increased H3K27ac (DBRs) in CHD2^+/-^ versus WT hcINs and that are also associated with a DEG with increased expression in CHD2^+/-^ versus WT hcINs.

**Supplemental Table S11. Quantitative PCR primers used in this study.**

**Supplemental Figure Legends**

**Supplemental Figure S1. Cluster analysis of gene expression changes during hMGE specification and hcIN differentiation.**

**(A)** Gene expression changes during specification of wild type hESCs as hMGE progenitors and their differentiation into hcINs. Only genes with a greater than two-fold change in gene expression (i.e. log2 RPKM > 1) from hESC-hMGE or from hMGE-hcIN are shown. Different colors indicate each gene cluster, as determined by K-means clustering based on fold changes. **(B-G)** Expression (RPKM) of key hMGE (NKX2.1, ASCL1, DLX2) and key hcIN genes (SST, GAD1, GAD2) from day 0-35 of hcIN differentiation.

**Supplemental Figure S2. Associations between CHD2 binding and risk genes for ASD or Epilepsy.**

Overlap of genes bound by CHD2 with high-confidence autism spectrum disorder (ASD)-associated genes (left Venn diagram and top heatmap) and epilepsy-related genes (right Venn diagram and bottom heatmap). Venn diagrams show numbers of genes bound by CHD2 in each cell state(s), with the center of the diagram showing the number of ASD/epilepsy-associated genes with at least one CHD2 binding event in hESCs, hMGEs, and hcINs. Heatmaps show expression (RPKM) of each gene in hESCs, hMGEs, and hcINs, color-coded and displayed in the order corresponding to the Venn diagram sections.

**Supplemental Figure S3. Gene network associated with CHD2 binding in hMGE progenitors.**

Gene network was generated from a single representative GO term (neuroepithelial development) associated with CHD2 binding unique to hMGE progenitors.

**Supplemental Figure S4. Gene expression changes of key transcription factors during hcIN differentiation.** Heatmaps of expression during hMGE specification and hcIN differentiation (RPKM) are shown for eight key classes of transcription factors that exhibited enriched transcription factor binding site motifs under CHD2 peaks in Homer analysis. CHD2 could function cooperatively or antagonistically with these transcription factors during hcIN differentiation.

**Supplemental Figure S5. Coenrichment of CHD2 bound peaks with several histone modifications.**

**(A**) deepTools plot showing all CHD2 bound peaks, and H3K4me3, H3K27ac, and H3K27me3 coenrichment at these CHD2 peak locations in hESCs, hMGEs, and hcINs. Chromatin state at regions bound by CHD2 in hESCs (top sections), in hMGEs (middle sections), and in hcINs (bottom sections) are shown separately. Plots at the top indicate the overall level of enrichment of each modification or CHD2 binding event. **(B)** Quantification of chromatin state at CHD2 peaks. Chromatin state at each CHD2 bound peak is indicated by the number of unique peaks for each histone modification that coenrich at sites of CHD2 binding at different stages of differentiation, considering all CHD2 bound peaks at each stage. CHD2 peaks indicated as 'bivalent' are coenriched for both H3K27me3 and H3K4me3. **(C)** Quantification of any CHD2 peaks that are coenriched for H3K4me3 or H3K27me3 at D0 and that are bivalent (e.g. coenriched for both histone modifications) at later time points, by comparison with the total number of CHD2-bound peaks that are coenriched for bivalent histone modifications across all time points (total bivalent). The peaks annotated only as D15/D35 bivalent are not enriched for H3K4me3 or H3K27me3 at D0.

**Supplemental Figure S6. Generation and analysis of hESCs heterozygous for a frameshift mutation in the *CHD2* gene.**

**(A)** Genetic sequence of the WT and mutant allele in the *CHD2^+/-^* hESC line, showing variant nucleotides in red for the WT and mutant allele. **(B)** Western blot analysis of CHD2 and GAPDH (loading control) levels in WT and *CHD2^+/-^* hESCs, with quantification at the left. **(C)** Karyotypic analysis of WT and *CHD2^+/-^* hESCs indicates a normal karyotype for both lines. **(D)** Comparison of the percentages of SST immunopositive hcINs in WT versus CHD2^+/-^ cultures after plating equivalent numbers of progenitors. n=3 biological replicate experiments. **(E)** Percentages of nuclei that were immunopositive for NKX2.1 in WT versus CHD2^+/-^ at D25 of differentiation. n=3 biological replicate experiments. **(F)** Representative images of D25 cells immunostained for NKX2.1. **(G)** Percentages of nuclei that were EdU immunopositive at D30, after receiving a 2 hr pulse of EdU at D20. **(H)** Representative images of EdU staining at D30, after a 2 hr pulse of EdU at D20. **(I)** Percentages of nuclei that were EdU immunopositive at D30 after receiving a 12 hr pulse of EdU at D20. **(J)** Representative images of EdU staining at D30, after cells received a 12 hr pulse of EdU at D20. Scale bar=100µm. Data is represented as mean ± SEM and was analyzed by an unpaired students t-test: **P<0.01 vs control. **(K-M)** Three replicate experiments, with the entire immunoblot shown, validate the finding shown in Fig. S6B (reduction of CHD2 levels upon disruption of one allele of the *CHD2* gene, results for two lines shown).

**References**

1 R: A language and environment for statistical computing v. 4.0.5 (2021-03-31) (R Foundation for Statistical Computing, Vienna, Austria, 2021).

2 Lambert, S. A. *et al.* The Human Transcription Factors. *Cell* **175**, 598-599, doi:10.1016/j.cell.2018.09.045 (2018).
